# Tumor-specific activatable biopolymer nanoparticles stabilized by hydroxyethyl starch prodrug for self-amplified cooperative cancer therapy

**DOI:** 10.7150/thno.67572

**Published:** 2022-01-01

**Authors:** Yuxuan Xiong, Zibing Wang, Qiang Wang, Qingyuan Deng, Jitang Chen, Jianshuang Wei, Xiaoquan Yang, Xiangliang Yang, Zifu Li

**Affiliations:** 1 National Engineering Research Center for Nanomedicine, College of Life Science and Technology, Huazhong University of Science and Technology, Wuhan, 430074, P. R. China; 2 Key Laboratory of Biomedical Photonics (HUST), Ministry of Education, Huazhong University of Science and Technology, Wuhan 430074, P. R. China; 3 Key Laboratory of Molecular Biophysics of Ministry of Education, College of Life Science and Technology, Huazhong University of Science and Technology, Wuhan, 430074, P. R. China; 4 Hubei Key Laboratory of Bioinorganic Chemistry and Materia Medical, Huazhong University of Science and Technology, Wuhan, 430074, P. R. China; 5 Hubei Engineering Research Center for Biomaterials and Medical Protective Materials, Huazhong University of Science and Technology, Wuhan, 430074, P. R. China; 6 GBA Research Innovation Institute for Nanotechnology, Guangdong, 510530, P. R. China; 7 Wuhan Institute of Biotechnology, High Tech Road 666, East Lake high tech Zone, Wuhan, 430040, P. R. China

**Keywords:** chemodynamic therapy, one-pot strategy, tumor-specific, GSH depletion, self-amplified therapy

## Abstract

**Rationale:** Chemodynamic therapy (CDT) is an emerging tumor-specific therapeutic strategy. However, the anticancer activity of CDT is impeded by the insufficient Fenton catalytic efficiency and the high concentration of glutathione (GSH) in the tumor cells. Also, it is challenging to eliminate tumors with CDT alone. Thus, simple strategies aimed at constructing well-designed nanomedicines that can improve therapeutic efficiency of CDT and simultaneously incorporate extra therapeutic modes as helper are meaningful and highly required.

**Method:** Tailored to specific features of tumor microenvironment (TME), in this study, we developed a biosafe, stable and TME-activated theranostic nanoplatform (P(HSD-Cu-DA)) for photoacoustic imaging (PAI) and self-amplified cooperative therapy. This intelligent nanoplatform was fabricated following a simple one-pot coordination and polymerization strategy by using dopamine and Cu^2+^ as precursors and redox-responsive hydroxyethyl starch prodrugs (HES-SS-DOX) as stabilizer.

**Results:** Interestingly, the pre-doped Cu^2+^ in polydopamine (PDA) framework can endow P(HSD-Cu-DA) NPs with tumor-specific CDT ability and remarkably enhance NIR absorption of PDA. PAI and biodistribution tests proved such nanoplatform can effectively accumulate in tumor tissues. Following enrichment, massive amounts of toxic hydroxyl radicals (·OH, for CDT) and free DOX (for chemotherapy) were generated by the stimulation of TME, which was further boosted by local hyperthermia. Concomitantly, in the process of activating these therapeutic functions, GSH depletion triggered by disulfide bond (-SS-) breakage and Cu^2+^ reduction within tumor cells occurred, further amplifying intratumoral oxidative stress. Importantly, the framework structure dominated by bioinspired polydopamine and clinical-used HES guaranteed the long-term biosafety of *in vivo* treatment. As a result, the mutual promotion among different components yields a potent tumor suppression outcome and minimized systemic toxicity, with one dosage of drug administration and laser irradiation, respectively.

**Conclusion**: This work provides novel insights into designing efficient and tumor-specific activatable nanotherapeutics with significant clinical translational potential for cancer therapy.

## Introduction

Currently, chemotherapy remains one of the mainstays of cancer treatment in clinical practice 1. But the unsatisfactory therapeutic efficacy and the adverse side effects arising from poor specificity to cancerous tissues limit the clinical success of chemotherapy 2-4. Developing tumor-specific nanomedicines that maximize therapeutic efficacy in tumor tissues with minimal side effects to healthy tissues is urgent yet challenging 5-7.

The tumor microenvironment (TME), which is featured with acidic pH, excessive hydrogen peroxide (H_2_O_2_), hypoxia and reducibility (intracellular), has been widely utilized to construct stimuli-driven and tumor-specific nanomedicines 8-10. In recent years, ROS-mediated chemodynamic therapy (CDT) is emerging as a promising therapeutic modality that leverages Fenton or Fenton-like reaction to convert endogenous H_2_O_2_ into highly toxic hydroxyl radicals (·OH) in the presence of metal catalysts (e.g., Fe, Cu, Mn, Co, etc.) 11-13. Attractively, CDT is considered as a tumor-specific therapeutic method because H_2_O_2_ is overproduced only within cancer cells 14, 15, while the acidic TME would accelerate the Fenton or Fenton-like reaction 16. This feature can drastically circumvent the toxicity of conventional chemotherapy towards normal tissues and cells. However, the relatively deficient catalytic efficiency restricts the yield of ·OH 17. Photothermal therapy (PTT) that employs hyperthermia to kill tumor cells has shown great potential in tumor-specific therapy due to its minimal invasiveness and spatiotemporal selective nature 18. Nevertheless, owing to the heterogeneous distribution of heat within tumor tissue, it is difficult to completely eradicate tumor cells by PTT alone. Further, tumor cells survived from PTT may lead to cancer recurrence and metastasis 19, 20. Theoretically, it is advisable to integrate PTT with CDT into a single nanoplatform since on one hand, it is reported that the hyperthermia generated from PTT could promote the catalytic efficiency of CDT 21, 22, and on the other hand, CDT would in turn kill residual tumors from PTT and reduce tumor recurrence and metastasis. Another serious problem of CDT is that the generated ·OH would be neutralized by the high concentration of glutathione (GSH) in cancer cells before inducing severe oxidative damage, discounting CDT 23. Hence, to reduce the unnecessary expenditure of ROS caused by the neutralization of GSH, nanomedicines with GSH-depleting ability are also desirable 24. Apparently, the realization of these claims is highly dependent on the optimal integration of nanomedicines. Up to now, there have indeed been some nanomedicines for integrating PTT/CDT and GSH depletion, but most of them are based on hard-to-degrade inorganic nanoparticles and involves in complex incorporation or assembly progress 25, 26, which are not conducive to clinical translation. Therefore, simple strategies aimed at constructing biocompatible and biodegradable nanomedicines that can improve catalytic efficiency of CDT via local hyperthermia and simultaneously decrease intracellular GSH, are of significant values.

Previous studies have shown that Cu^+^-catalyzed Fenton-like reactions can occur efficiently in weakly acidic and neutral media, while their reaction rates are much higher than those conventional Fe-based Fenton or Fenton-like reactions 27, 28. Owing to the unstable nature of Cu^+^, Cu^2+^ is generally employed as CDT reagent because it can be reduced to Cu^+^ locally by overexpressed GSH within tumor cells 29. However, excess free Cu^2+^ may produce severe systemic toxicity 30, so it is important to specifically deliver Cu^2+^ to tumor tissues while avoid burst release in circulating and normal tissues. Melanin-like polydopamine (PDA) is a promising candidate for efficient PTT due to its excellent biocompatibility and biodegradability 31. Interestingly, PDA possesses the ability to immobilize metal ions (e.g., Cu^2+^, Fe^3+^, Mn^2+^) through coordination interactions 32, 33. This allows PDA not only for PTT but also as an ideal carrier for Cu^2+^. More encouragingly, studies show that the doping of Cu^2+^ can significantly enhance the NIR absorption of PDA 34, 35, which is favorable for PTT. Nevertheless, pure PDA is unstable under physiological conditions. Our previous work has demonstrated that hydroxyethyl starch (HES), a clinical used plasma volume expander with excellent biocompatibility, biodegradability and well-defined* in vivo* safety, can greatly improve the stability of PDA in physiological environment 36. In addition, to overcome the side effects of chemotherapeutic drugs, many HES-based, TME-responsive prodrugs have been prepared by our lab 37, 38. We assume that some of these HES prodrugs can also stabilize PDA while achieve tumor-specific chemotherapeutic effects which is a decent complement to CDT.

**Scheme 1:**
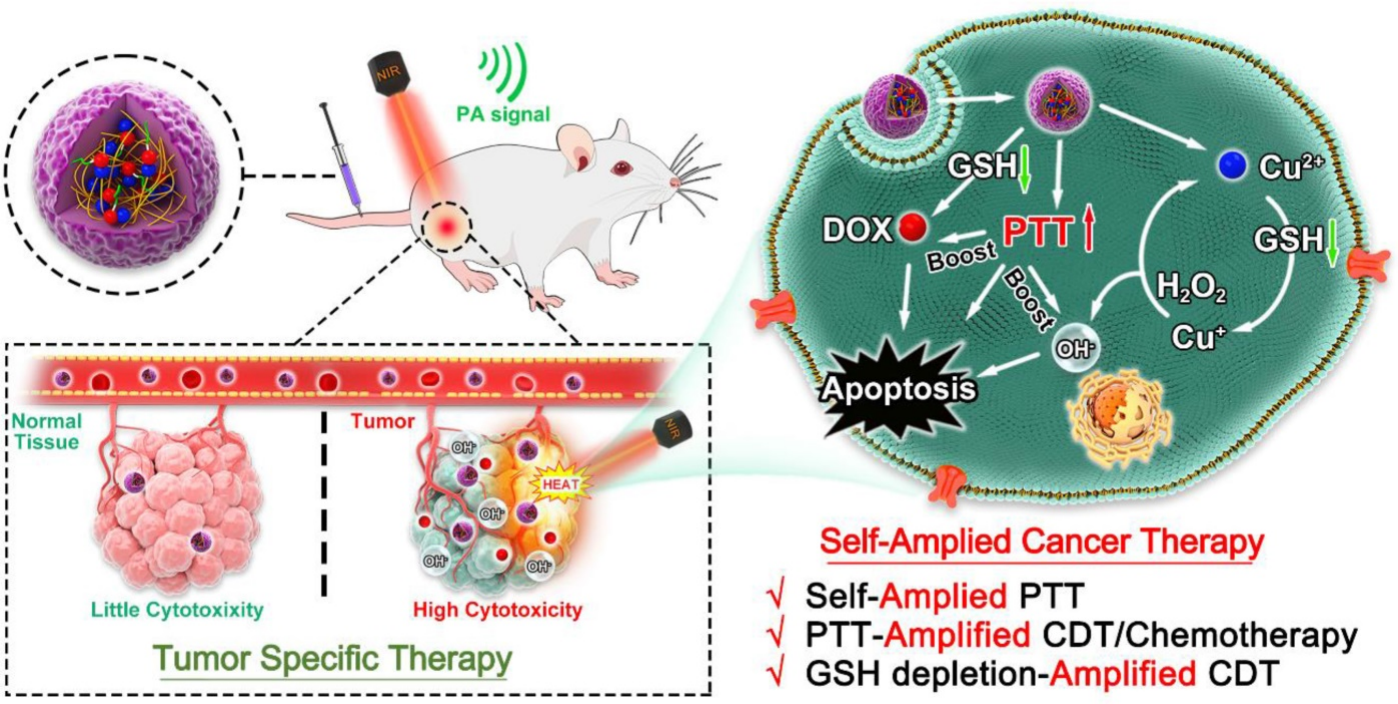
Schematic illustration of P(HSD-Cu-DA) NPs for self-amplified tumor-specific therapy.

With this, we present a simple one-pot coordination and polymerization strategy to synthesize multifunctional copper-enriched polydopamine nanoparticles (denoted as P(HSD-Cu-DA) NPs) under the stabilization of HES-SS-DOX (HSD, a redox-sensitive prodrug of doxorubicin (DOX) prepared in our lab 37) for efficient tumor-specific therapy (**Scheme 1**). HSD not only acts as a stabilizer for nanoparticles but also provides additional tumor-specific chemotherapy. Impressively, Cu^2+^ plays triple roles in P(HSD-Cu-DA) NPs-mediated cancer-specific therapy. First, Cu^2+^ is introduced as “bridge” for coordination and confers P(HSD-Cu-DA) NPs with CDT capability. Second, Cu^2+^ with oxidizing properties serves as a GSH-depleting agent. Third, Cu^2+^ pre-doping greatly enhances the NIR absorption of PDA. Moreover, the excellent photothermal ability in turn promotes the catalytic ability of Cu^2+^ and the TME stimuli-responsive release of DOX. Additionally, not only the reduction of Cu^2+^ but also the breakage of disulfide bonds within P(HSD-Cu-DA) NPs can provoke a decline in intracellular GSH, attenuating the unnecessary ROS loss. All these active ingredients in P(HSD-Cu-DA) NPs collaborate with each other and work selectively in tumor TME. Consequently, the established P(HSD-Cu-DA) NPs exhibit good biocompatibility and powerful ability for tumor-specific cooperative therapy, offering great potential for clinical translation.

## Results and Discussion

### Preparation and Characterization of P(HSD-Cu-DA) NPs

**Figure 1:**
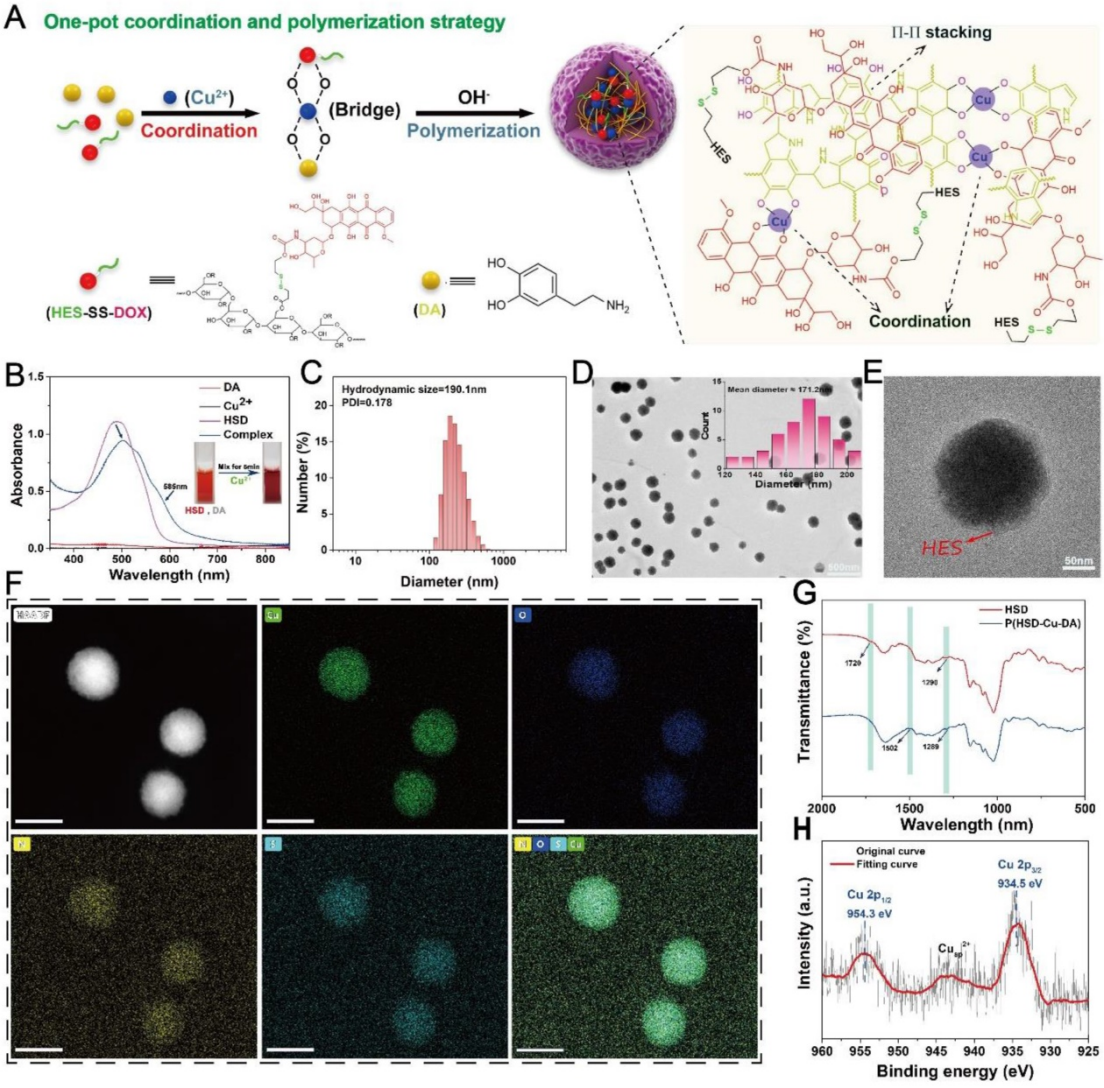
** Chemical structure confirmation of P(HSD-Cu-DA) NPs.** (A) Schematic diagram of the formation of P(HSD-Cu-DA) NPs. (B) Absorption spectra and digital images (inset) before and after coordination with Cu^2+^. (C) Particle diameter of P(HSD-Cu-DA) NPs. (D) (E) TEM images of P(HSD-Cu-DA) NPs at different magnifications. The inserted figure is the size statistics of NPs in TEM image. Scale bar is 500 nm in (D) and 50 nm in (E), respectively. (F) Elemental mapping of P(HSD-Cu-DA) NPs. Scale bars are 200 nm. (G) FTIR spectra of HSD and P(HSD-Cu-DA). (h) XPS high-resolution scan of Cu 2p in P(HSD-Cu-DA) NPs.

The impact of Cu^2+^ incorporation on NIR absorption of polydopamine (PDA) was initially studied. Keeping dopamine content constant (2 mM), the absorption intensity (NIR region) of P(Cu-DA) was found to improve significantly with the increase of doped Cu (**Figure S1A**). This phenomenon may be attributed to that the pre-doping of copper ions accelerates the polymerization of dopamine, leading to higher reaction yields 34, 35, 39. When the molar ratio for Cu and dopamine exceeded 1:1, a visible precipitate formed rapidly. Therefore, this Cu/dopamine ratio (1:1) was adopted for subsequent procedures. The versatile P(HSD-Cu-DA) NPs were synthesized by a facile “one pot” strategy, as illustrated in **Figure 1A**, utilizing HES prodrug (HSD) as stabilizer (prepared by our lab). Copper ion was employed as “bridge” for connecting dopamine and HSD *via* coordination effect. HSD can also be attached onto PDA framework by π-π stacking or electrostatic interactions 40, 41. After adding Cu^2+^ to the mixture, the absorbance at 485 nm decreased, while the absorbance at 585 nm increased. At the same time, the color of the mixture quickly changed from red (DOX) to purple (**Figure 1B**). These facts confirmed that DOX in HSD did chelate with Cu^2+^ 42, 43. When the reaction system was adjusted to alkalinity, polymerization occurred. The characteristic absorption peak of DOX gradually intensified as the HSD feeding ratio increased (**Figure S1B**). It is worth noting that the HSD played a critical role in modulating the particle size of P(HSD-Cu-DA). A feeding ratio of 1:1:0.3 (DA: Cu: DOX) was determined for subsequent experiments as the final nanoparticles obtained with this ratio had a more uniform size distribution (Hydrodynamic diameter=190.1 nm, PDI=0.178) relative to other feeding ratios (**Figure S1C**).

The detailed morphology of P(HSD-Cu-DA) NPs was characterized by transmission electron microscopy (TEM), **Figure 1D**. The mean diameter of the NPs was around 171.2 nm, which was slightly smaller than the value obtained from DLS measurements (**Figure 1C**). A thin layer on the surface of P(HSD-Cu-DA) NPs was observed under high magnification TEM (**Figure 1E**), which may be attributed to the hydrophilic HES. Meanwhile, the uniform element distribution of Copper (Cu), Oxygen (O), Nitrogen (N) and Sulphur (S) in **Figure 1F** indicated the successful formation of P(HSD-Cu-DA) NPs. To further assess the structure of P(HSD-Cu-DA) NPs, Fourier Transform Infrared (FTIR) spectra of HSD and P(HSD-Cu-DA) NPs were carefully investigated. As shown in **Figure 1G**, the characteristic bands of HSD at 1290 cm^-1^ and P(HSD-Cu-DA) NPs at 1289 cm^-1^ were both ascribed to C-N stretch vibration of the amide bonds in HSD 37. A new peak occurred at 1502 cm^-1^ was assigned to the shearing vibration of N-H of PDA 44. In contrast to HSD, the characteristic peak of carbonyl groups (1720 cm^-1^) disappeared for P(HSD-Cu-DA) NPs, suggesting the coordination of Cu to carbonyl groups 27, 45. Moreover, the chemical valence of Cu on the surface of P(HSD-Cu-DA) NPs was determined by X-ray Photoelectron Spectroscopy (XPS). As represented in **Figure 1H**, the high-resolution spectra of Cu 2p displayed two main peaks at 934.5 eV (Cu 2p_3/2_) and 954.3 eV (Cu 2p_1/2_), coupled with the satellite peak at 944.2 eV, confirming the existence of Cu(Ⅱ) in P(HSD-Cu-DA) NPs 46. In addition, N 1s and S 2p were found in the survey spectrum (**Figure S2A**), further evidencing the presence of dopamine and HSD 47. The Cu content in P(HSD-Cu-DA) NPs was identified as ~1.81% (mass%) by ICP-OES, while the DOX content in P(HSD-Cu-DA) NPs was calculated to be ~3.64% (mass%). The zeta potential of P(HSD-Cu-DA) NPs was around -10.69 mV as compared to -19.3 mV for P(Cu-DA) NPs (**Figure S2B**). This apparent change in surface charge further indicted the successful coordination of HSD with Cu in P(HSD-Cu-DA) NPs. This negatively charged NPs also facilitated long-lasting blood circulation of nanomedicines 48. The colloidal stability of the NPs was assessed prior to subsequent characterization. No obvious change (<20%) in nanoparticle size was observed after one week of storage in different media (PBS, 10% FBS, and saline) (**Figure S3A**). Additionally, the long-term stability of P(HSD-Cu-DA) and P(Cu-DA) NPs in PBS was also examined in comparison. It was found that P(HSD-Cu-DA) NPs remained stable even after one month storage, whereas a visible precipitation appeared at the bottom of the P(Cu-DA) solution (**Figures S3B-C**), highlighting the stabilizing effect of HSD.

### Functional characterization of P(HSD-Cu-DA) NPs

**Figure 2:**
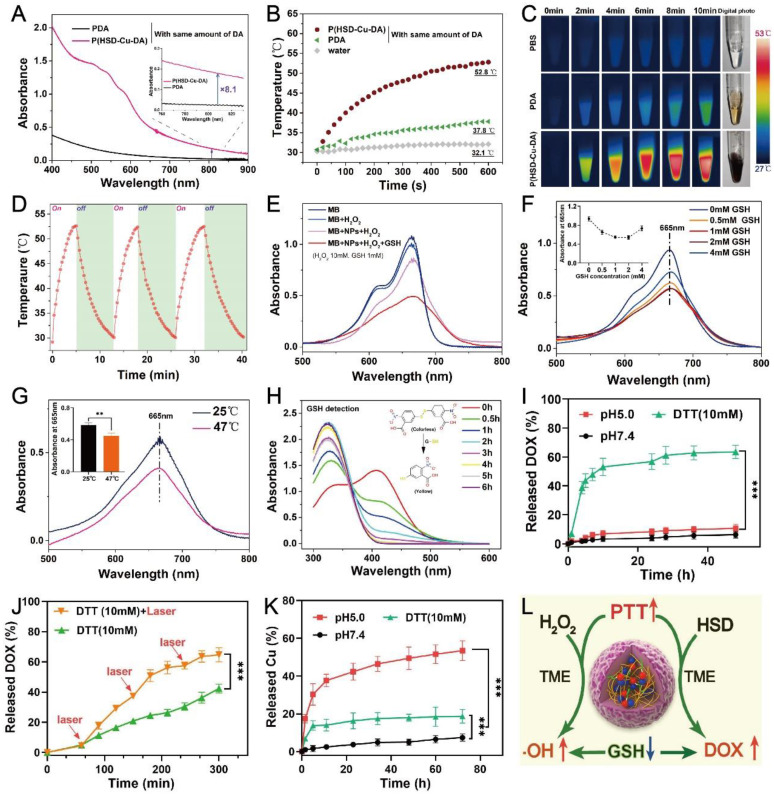
** Functional characterization of P(HSD-Cu-DA) NPs.** (A) Absorption spectra of PDA and P(HSD-Cu-DA) NPs with same amount of DA monomer. The inset figure shows a magnified absorption spectrum. Temperature elevation curves (B) and corresponding photothermal images (C) of PDA and P(HSD-Cu-DA) upon NIR irradiation (808 nm, 1W/cm^2^). (D) Photothermal stability of P(HSD-Cu-DA) NPs under 808 nm laser (three times laser on/off). (E) ·OH-induced MB degradation after different treatments. (F) MB degradation by ·OH generated from P(HSD-Cu-DA) NPs treated with different concentrations of GSH. The inset figure is a statistical analysis. (G) Photothermally enhanced ·OH generation of P(HSD-Cu-DA) NPs. The inset figure shows the quantitative results. (H) GSH depletion at different time points with DTNB as detecting probe. The inserted image is the detection mechanism of DTNB. (I) Accumulative release of DOX from P(HSD-Cu-DA) NPs at different medium. (J) Photothermal-enhanced DOX release. (K) Release profile of Cu from P(HSD-Cu-DA) NPs at different medium. (L) The overall scheme of P(HSD-Cu-DA) NPs for self-amplified therapy. **, P < 0.01; ***, P < 0.001.

The photothermal capacity of P(HSD-Cu-DA) NPs was assessed. Firstly, the absorption intensity of P(HSD-Cu-DA) NPs in the NIR region was measured. Keeping the dopamine (DA) monomer masses and reaction conditions consistently, the absorption of P(HSD-Cu-DA) NPs solution at 808 nm was 8.1 times higher than that of PDA NPs (**Figure 2A**), which was ascribed to the pre-doped Cu^2+^. Meanwhile, the absorption peak between 480-600 nm corresponded to DOX. Owing to the strong absorption in NIR region, the photothermal ability of P(HSD-Cu-DA) NPs was investigated under NIR laser irradiation at different concentrations. As indicated in **Figure S4A**, the photothermal effect was found to be time and concentration dependent. Following NIR irradiation (1 W/cm^2^), P(HSD-Cu-DA) NPs solution can elevate 22.4 °C in 10 min, whereas PDA NPs solution with the same amount of dopamine only raised the temperature by 7.1 °C (**Figure 2B-C**). Such marked difference in temperature enhancement was consistent with the results of the absorption spectra. The photothermal conversion efficiency (η) of P(HSD-Cu-DA) NPs was calculated from the fitted cooling curve to be 40.9%, which was comparable to that of PDA (41.4%) (**Figures S4B-C**). The influence of HSD on the photothermal performance of P(HSD-Cu-DA) NPs was further studied. As exhibited in **Figure S5**, after the introduction of HSD, the absorption of P(HSD-Cu-DA) in the NIR region (700-850 nm) did not change significantly relative to P(Cu-DA). Interestingly, P(HSD-Cu-DA) showed a higher temperature elevation than P(Cu-DA) (55.1°C vs 51.2°C) after irradiation with the same power of NIR light, which may be due to the weaker stability of P(Cu-DA) NPs. Moreover, three on/off cycles of laser irradiation (**Figure 2D**) revealed the laudable photothermal stability of P(HSD-Cu-DA) NPs. Given its favorable thermal stability and high η, the potential of P(HSD-Cu-DA) NPs as photothermal agents in tumor therapy is foreseeable.

The pre-incorporation of Cu^2+^ not only significantly enhanced NIR absorption than pure PDA, but the reduction of Cu^2+^ allowed for the depletion of GSH within tumor cells, and the subsequently produced Cu^+^ can react with endogenous H_2_O_2_ to produce highly toxic ·OH via a Fenton-like reaction, which was essential for CDT 27. Here, ·OH-generating performance of P(HSD-Cu-DA) NPs was evaluated adopting methylene blue (MB) degradation assay. As depicted in** Figure 2E**, H_2_O_2_ alone cannot noticeably alter the MB absorbance. In contrast, after co-incubation with P(HSD-Cu-DA) NPs ([Cu]=1mM) for 2 h, MB was apparently degraded. Unfortunately, the MB degradation rate (~18%) remained limited. Interestingly, the degradation rate (55%) of MB was significantly boosted by the introduction of GSH (1mM). The impact of GSH concentration on MB degradation was further studied. The results (**Figure 2F**) revealed that the MB degradation rate exhibited a trend of increase followed by decrease with increasing GSH concentration (0-4mM). This was because moderate GSH could promote the conversion of Cu^2+^ to Cu^+^, which favored the production of ·OH, whereas excessive GSH would neutralize some ·OH, leading to a decrease of MB degradation rate. Previous studies indicated that hyperthermia can promote the catalytic activity of CDT 23, 49. Considering the excellent photothermal effect of P(HSD-Cu-DA) NPs, we gave further verification whether photothermal can promote the production of ·OH. As illustrated in **Figure 2G**, the MB degradation efficiency was distinctly reinforced by NIR irradiation (plateau temperature: 47°C). These data suggested that P(HSD-Cu-DA) NPs had the capability to achieve robust CDT against tumors.

Overexpressed GSH within cancer cells has been reported to impede cancer therapy. Depleting GSH within cancer cell was thus considered a potent solution to improve the therapeutic outcome 24. For the present nanomedicine, although GSH was involved in the reduction of Cu^2+^, which was crucial to ·OH production, too much GSH was still a hindrance to CDT and therefore GSH depletion was necessary. The GSH depletion capacity of P(HSD-Cu-DA) NPs was investigated using Ellman's reagents by the mechanism that colorless DTNB can be reduced by GSH to form a yellow product (absorbance at 412 nm) 50. As shown in **Figure 2H**, with the addition of P(HSD-Cu-DA) NPs ([Cu]=1mM), the absorbance intensity at 412 nm decreased sharply with elongated time. After 6 h of incubation, the absorption peak at 412 nm disappeared, indicating that the GSH (1mM) in this reaction system was entirely consumed. These results demonstrated the potent GSH-depleting ability of P(HSD-Cu-DA) NPs, which was largely attributed to the reduction of Cu^2+^ 27 and the breaking of disulfide bonds 51. Besides, the size and morphology of P(HSD-Cu-DA) NPs after GSH (10mM) treatment were also characterized. As shown in **Figure S6**, the hydrodynamic diameter of P(HSD-Cu-DA) NPs increased significantly from ~190.1nm to ~1246.0nm after incubation with GSH for 48h. Corresponding TEM images further confirmed the aggregation of P(HSD-Cu-DA) NPs in the presence of GSH. This may be due to the fact that GSH severed the connection between HES and PDA framework, resulting in decreased stability of nanoparticles. This aggregation property may favor the retention of nanomedicine in the tumor tissues 52.

The release of DOX from P(HSD-Cu-DA) NPs was assessed in both reducing and acidic conditions. **Figure 2I** showed that only trace amounts of DOX were released in the pH 5.0 (~10.59%) and pH 7.4 (~6.22%) environment during 48 h. In sharp contrast, the supplementation with 10 mM of dithiothreitol (DTT, a frequently-used GSH analogue as the reductant) triggered more than 60% of DOX release within 48 h. The fluorescence recovery experiments of DOX also confirmed the reduction-responsive drug release behavior (**Figure S7**). This tumor-specific release behavior was attributed to the existence of disulfide bonds in P(HSD-Cu-DA) NPs. The influence of hyperthermia on DOX release was further investigated. As displayed in **Figure 2J**, after exposure to an 808 nm NIR laser (1 W/cm^2^, 10 min for each pulse), the cumulative DOX release reached 64.6% compared to 42.3% without NIR irradiation within 5 h. Similar photothermally enhanced DOX release behavior was also observed in the presence of GSH (**Figure S8**). Such differences may be explained by the fact that NIR-induced hyperthermia promoted the breakage of disulfide bonds while weakening the interaction between the DOX and PDA frames 53, 54. The excellent stability of P(HSD-Cu-DA) NPs ensured that it did not release the drug prematurely in the circulation and normal tissues, while its reduction-responsive and NIR-enhanced drug release behavior allowed it to specifically dump more DOX within cancer cells.

Although Cu is an essential trace element for the human body, excessive Cu exposure can be highly toxic and cause many diseases 30. Hence, the stability of Cu in P(HSD-Cu-DA) NPs in different release media was carefully studied. The results (**Figure 2K**) showed that only a minimal amount of Cu^2+^ (~7.38%) was released from P(HSD-Cu-DA) NPs at pH 7.4 even if the release time was extended to 72 h, indicating that they were stable in a physiological environment. Interestingly, the presence of DTT (10 mM) or in pH 5.0 condition accelerated the release of Cu^2+^, with 18.6% and 53.4% released after 72 h incubation, respectively. This phenomenon was probably triggered by the breakage of the Cu-O bond under acidic conditions 55, 56. These results suggested that Cu was liberated in a high level under acidic or reductive tumor conditions whereas leakage was minimal under normal physiological conditions, which is a boon for tumor-specific therapy. In addition, the photothermal performance of P(HSD-Cu-DA) NPs under different pH conditions was evaluated. As shown in **Figure S9**, the NIR absorption intensity and photothermal effect of P(HSD-Cu-DA) NPs did not change significantly after incubation at pH 5 for 48 h, indicating that the release of some copper ions had a small effect on the photothermal effect of P(HSD-Cu-DA) NPs. We speculate that this may be because, Cu^2+^ only promoted the polymerization of DA and increased the yield of PDA, while Cu ions itself had little effect on the NIR absorption.

The self-amplified mechanism of P(HSD-Cu-DA) NPs were summarized as follows, **Figure 2L**. (1) The coordination of Cu^2+^ dramatically facilitated NIR absorption of PDA, resulting in a better photothermal effect (**Figure 2A-C**). (2) In turn, this reinforced photothermal effect boosted the generation of highly toxic ·OH (**Figure 2G**) and the release of chemotherapeutic drug DOX (**Figure 2J**). (3) GSH depletion (**Figure 2H**) induced by Cu^2+^ reduction and disulfide bond breakage additionally promoted CDT by lessening unnecessary ·OH loss. We would further validate this self-amplified therapeutic modality in subsequent experiments both *in vitro* and *in vivo*.

### *In vitro* ROS generation and GSH depletion by P(HSD-Cu-DA) NPs

**Figure 3:**
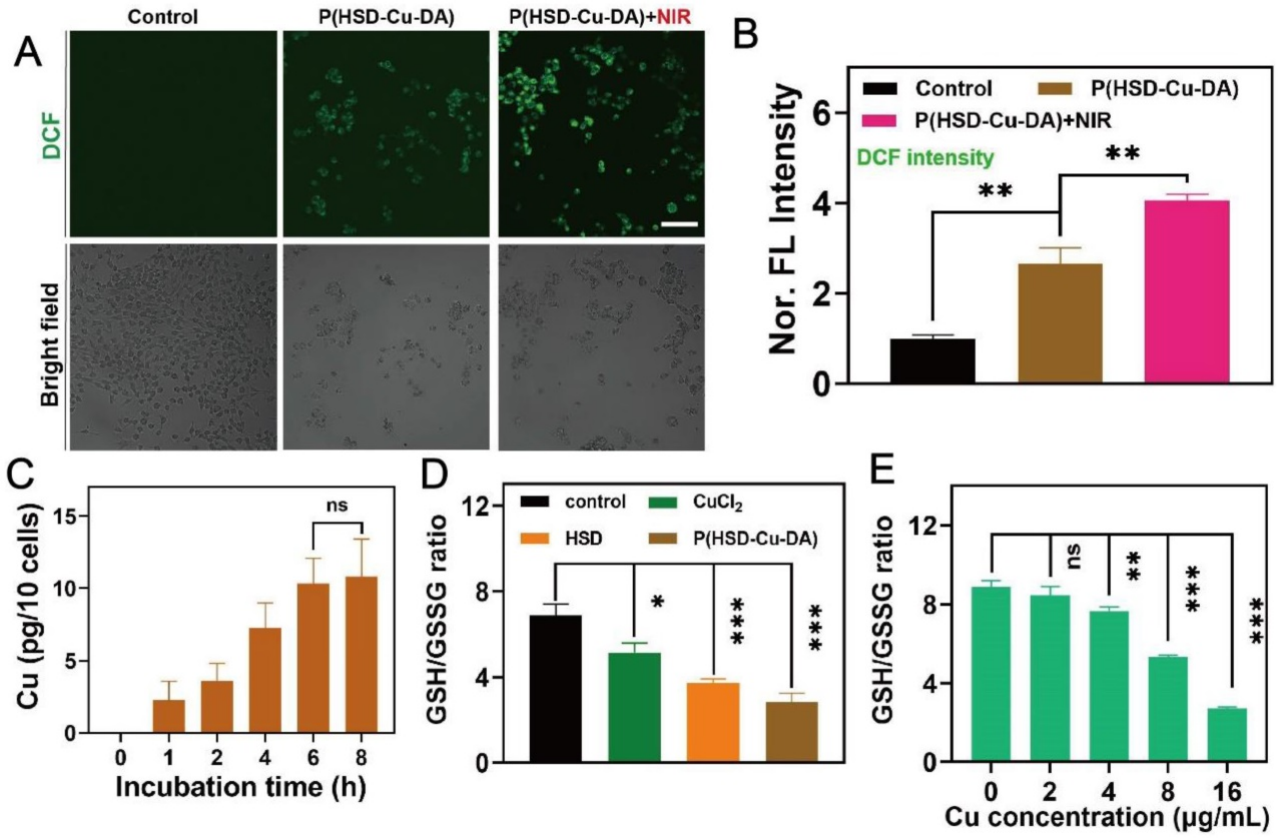
** Intracellular redox environment modulation with P(HSD-Cu-DA) NPs.** CLSM images (A) and corresponding fluorescence quantification (B) of 4T1 cells staining with DCFH-DA after different treatments. Scale bar is 100 μm. (C) Cellular uptake of P(HSD-Cu-DA) NPs by quantifying Cu content using ICP-OES. (D) GSH/GSSG ratio in 4T1 cells after different treatments. (E) GSH/GSSG ratio of 4T1 cells treated with different concentrations of P(HSD-Cu-DA) for 6 h. ns stands for not significant; *, P < 0.05; **, P < 0.01; ***, P < 0.001.

To confirm that P(HSD-Cu-DA) NPs can be effectively taken up by cancer cells, we firstly investigated the uptake of P(HSD-Cu-DA) NPs by 4T1 cells with ICP-OES. As seen in **Figure 3C**, the cellular uptake reached a plateau after 6 h incubation. Probe 2',7'-dichlorofluorescin diacetate (DCFH-DA), which can be oxidized to fluorescent 2',7'-dichlorofluorescein (DCF) in cells 57, was used to evaluate intracellular oxidative stress. As expected, 4T1 cells treated with Cu^2+^ showed stronger green fluorescence compared to the control group, probably owing to the conversion of endogenous H_2_O_2_ to ·OH with the assistance of Fenton-like reaction of Cu^2+^ (**Figures S10**). The influence of heat on ROS production was further studied. As shown in **Figure 3A-B**, after 5 mins of NIR exposure, the oxidative stress induced by P(HSD-Cu-DA) NPs was increased by 1.5 folds of that in the non-irradiated group, indicating that the photothermal effect was capable of hastening the production of ·OH. This result was in accordance with the MB degradation experiments described above.

Subsequently, intracellular GSH consumption induced by P(HSD-Cu-DA) NPs was evaluated by a GSH and GSSG Assay Kit. GSH/GSSG ratio was a reflection of intracellular redox status 58. Both Cu^2+^ and HSD were found to reduce the GSH/GSSG ratio, and the effect of HSD was surprisingly more pronounced, which was presumably due to the large number of disulfide bonds in HSD 59. Under the blessing of Cu^2+^ and HSD, both GSH/GSSG ratio and GSH content were greatly decreased by P(HSD-Cu-DA) NPs (**Figure 3D, Figure S11**), indicating an increase in intracellular oxidation levels, which was mainly attributed to GSH depletion and ·OH production. Meanwhile, GSH/GSSG ratio showed a clear Cu concentration dependence (**Figure 3E**). Collectively, these results demonstrate that P(HSD-Cu-DA) NPs was able to amplify oxidative stress at the cellular level by generating ·OH and depleting intracellular GSH, which prompted us to perform subsequent cytotoxicity experiments.

### *In vitro* antitumor efficiency of P(HSD-Cu-DA) NPs

**Figure 4:**
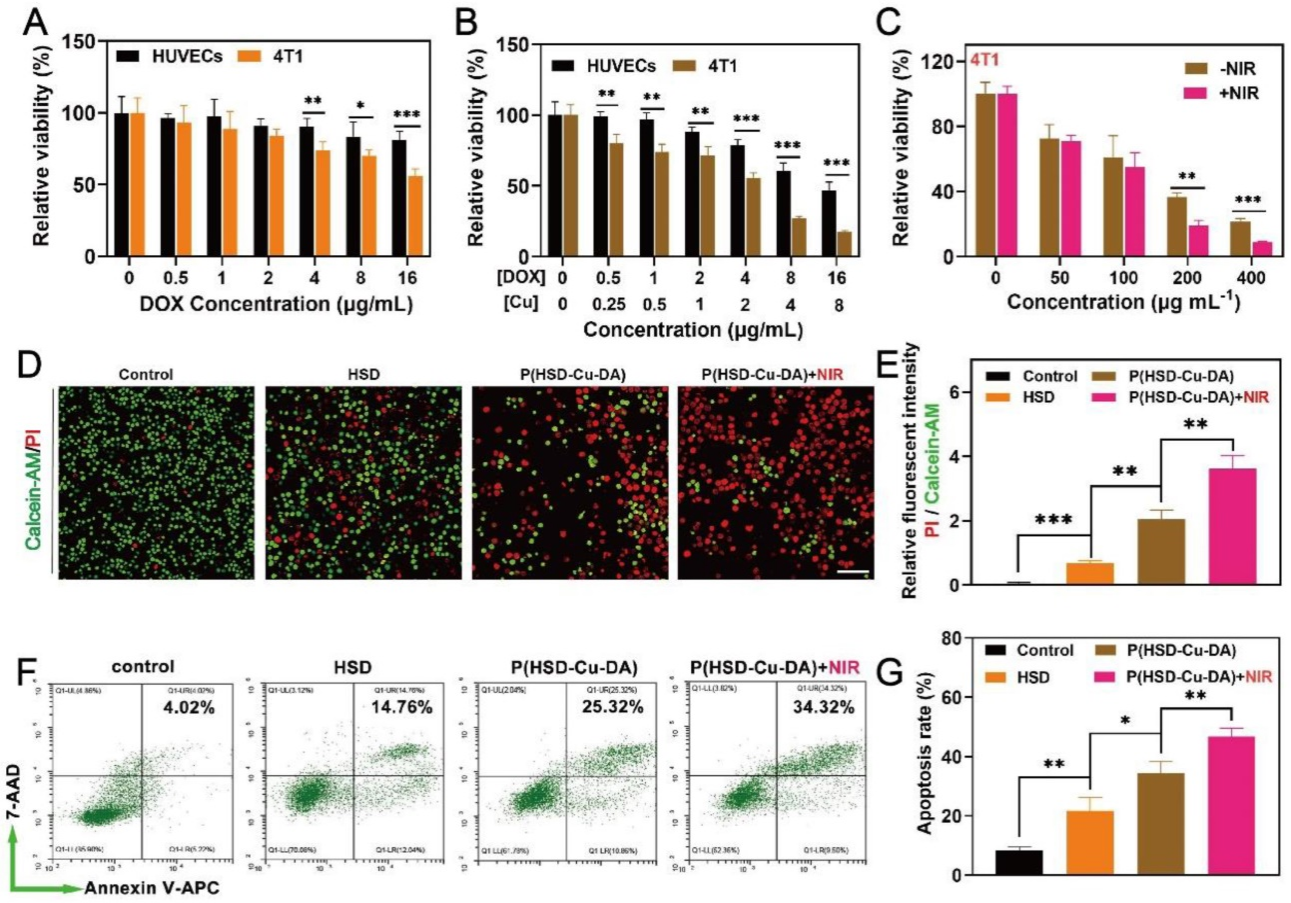
***In vitro* anti-tumor activity of P(HSD-Cu-DA) NPs.** Cell survival of 4T1 cells (A) and HUVECs (B) with different concentrations of HSD and P(HSD-Cu-DA) NPs. (C) Cell survival of 4T1 cells cultured with different concentrations of P(HSD-Cu-DA) NPs with / without 808 nm irradiation. (D) Calcein-AM and PI staining images and (E) their relative fluorescent intensity in 4T1 cells after different treatments. Scale bar is 100μm. (F) Cell apoptosis assay by flow cytometry in 4T1 cells with Annexin V-APC and 7-AAD double staining, and corresponding quantitative analysis shown in (g). *, P < 0.05; **, P < 0.01; ***, P < 0.001.

Encouraged by the oxidative stress induced by P(HSD-Cu-DA) NPs, we next assessed the potential cytotoxicity of the NPs on both normal and cancer cells by 3-(4,5-dimethyl-2-thiazolyl)-2,5-diphenyl-2-H-tetrazolium bromide (MTT) assay. We first evaluated the inhibition effect of HSD on 4T1 tumor cells and normal human umbilical vein endothelial cell (HUVECs). As depicted in **Figure 4A**, HSD showed a concentration-dependent killing tendency towards 4T1 tumor cells, but had negligible effects on HUVECs, especially at high concentrations of 4-, 8-, and 16-μg/mL of DOX. The cytotoxicity of P(HSD-Cu-DA) NPs was also assessed using these two cell lines. Distinctly, the cytotoxicity was universally higher against 4T1 cells than HUVECs cells at various concentrations, with half maximal inhibitory concentration (IC50) values of 4.34 and 13.50 μg/mL (DOX), respectively (**Figure 4B**). Meanwhile, remarkable cell killing was also observed on both Panc02 and B16-F10 cells, with IC50 values of 6.83 and 3.85 μg/mL (DOX), respectively (**Figure S12**). This tumor-specific killing behavior was mainly attributed to the different intracellular environments. It was reported that the concentration of GSH in tumor cells was at least four times higher than that of normal cells 24, which on the one hand favored the release of DOX, and on the other hand P(HSD-Cu-DA)-induced GSH depletion promoted intracellular ROS accumulation. In addition, a cross-sectional comparison revealed that the cytotoxicity of P(HSD-Cu-DA) NPs was significantly stronger than that of HSD at the same DOX concentration, which was attributed to Cu^2+^-mediated enhanced oxidative stress. Given its excellent NIR absorption and photothermal performance, photothermally enhanced cytotoxicity of P(HSD-Cu-DA) NPs was further studied. As shown in **Figure 4C**, upon irradiation, the cell killing efficiency was significantly increased. This enhancement effect is more prominent at higher concentrations because of the higher temperature rise.

To visually evaluate the cytotoxicity on 4T1 cells, the live cells were stained green by Calcein-AM and the dead cells were stained red by propidium iodide (PI). Consistently, the P(HSD-Cu-DA) NPs plus laser irradiation group resulted in the highest cell mortality (**Figure 4D-E**). Furthermore, the lethal mechanism was analyzed by flow cytometry. To avoid the interference from DOX fluorescence, Annexin V-APC/7-AAD apoptosis kit was adopted. As seen in **Figure 4F**-**G**, P(HSD-Cu-DA) NPs plus laser treatment elicited a higher apoptosis rate in 4T1 cells (46.73%, sum of Annexin V-APC^+^ / 7AAD^+^ and Annexin V-APC^+^ / 7AAD^-^) compared with PBS (8.48%), HSD (21.72%) and P(HSD-Cu-DA) NPs (34.45%) treated groups, suggesting the apoptosis-induced cell death pathway. All the above results demonstrated the favorable anti-tumor efficacy of P(HSD-Cu-DA) NPs in* in vitro* conditions.

### Pharmacokinetics and PA imaging of P(HSD-Cu-DA) NPs

**Figure 5:**
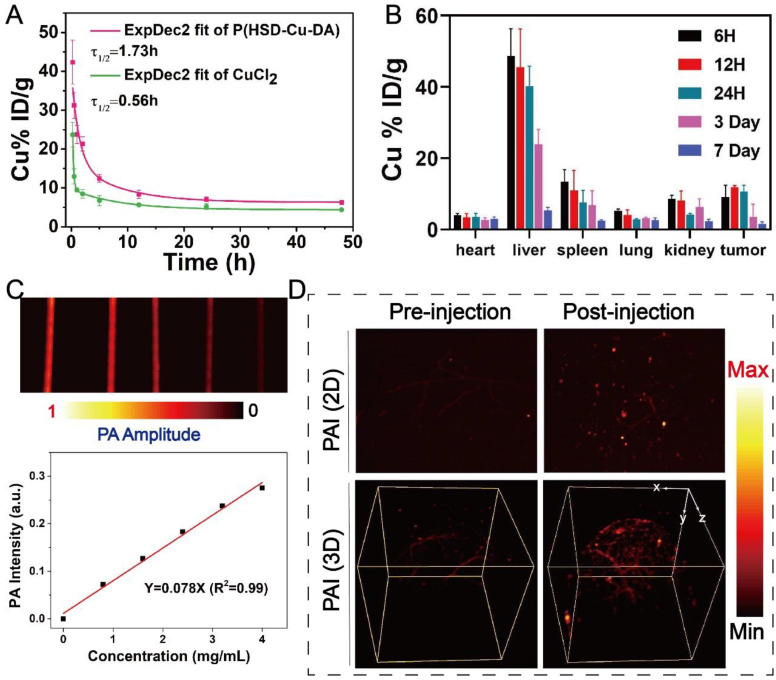
***In vivo* analysis of P(HSD-Cu-DA) NPs.** (A) Pharmacokinetics of CuCl_2_ and P(HSD-Cu-DA) NPs in blood circulation determined by ICP-OES. (B) Biodistribution of Cu in the major organs and tumors at different time points post injection of P(HSD-Cu-DA) NPs. (C) *In vitro* PAI of different concentrations of P(HSD-Cu-DA) NPs. (D)* In vivo* 2D and 3D PA tumor imaging before and 12 h after vein injection of P(HSD-Cu-DA) NPs.

In light of the positive results from *in vitro* studies, the biological application of P(HSD-Cu-DA) NPs was further examined in mice. The *in vivo* behavior of P(HSD-Cu-DA) NPs was first investigated prior to subsequent imaging and pharmacodynamic evaluation. The circulating half-life of P(HSD-Cu-DA) NPs in the blood stream was calculated as 1.73 h by a double compartment pharmacokinetic model, which was more than three times that of free CuCl_2_ (**Figure 5A**). This may be related to the stabilizing effect of HES in P(HSD-Cu-DA) NPs 36. In addition, the biodistribution of P(HSD-Cu-DA) NPs in tumors and major organs at various time points after intravenous administration was investigated by ICP-OES. Endogenous copper contents in tumors and major organs of blank mice were first measured (**Figure S13**). As shown in **Figure 5B**, the massive accumulation of NPs in the liver and spleen was mainly due to the uptake by the reticuloendothelial system (RES) 60, whereas the enrichment in the kidney may be due to secretion by tubule epithelial cells from the peritubular capillaries 61. It was found that P(HSD-Cu-DA) NPs can effectively accumulate in tumors by EPR effect, with relative enrichment rate as high as ~8.87% at 6 h, increasing to ~11.72% at 12 h, followed by a gradual decline. So 12 h post injection was chosen as the desired time point for testing and irradiation. 7 days after injection, only a small number of NPs remained in the major organs, which avoided long-term toxicity to some extent. Given the strong NIR absorption of P(HSD-Cu-DA) NPs, we envisioned that it could achieve superior PA-imaging performance. The PA imaging ability of P(HSD-Cu-DA) NPs was tested both *in vitro* and *in vivo*. As revealed in **Figure 5C**, the signal intensity of PA was linearly enhanced with increasing concentration of P(HSD-Cu-DA) NPs under excitation with a 744 nm laser. *In vivo* PA imaging was conducted 12 h after tail vein injection of P(HSD-Cu-DA) NPs. Compared to blank control, a markedly stronger PA signal was observed in both 2D and 3D PA images of NPs-treated mice (**Figure 5D**), suggesting P(HSD-Cu-DA) NPs accumulated within tumor tissues.

Taken together, these results indicated that P(HSD-Cu-DA) NPs can effectively enrich in tumor tissues by EPR effects. Meanwhile, the satisfactory PA imaging performance of P(HSD-Cu-DA) was confirmed, which had important implications for pre- and post-treatment *in vivo* monitoring.

### *In vivo* therapeutic efficacy of P(HSD-Cu-DA) NPs

**Figure 6:**
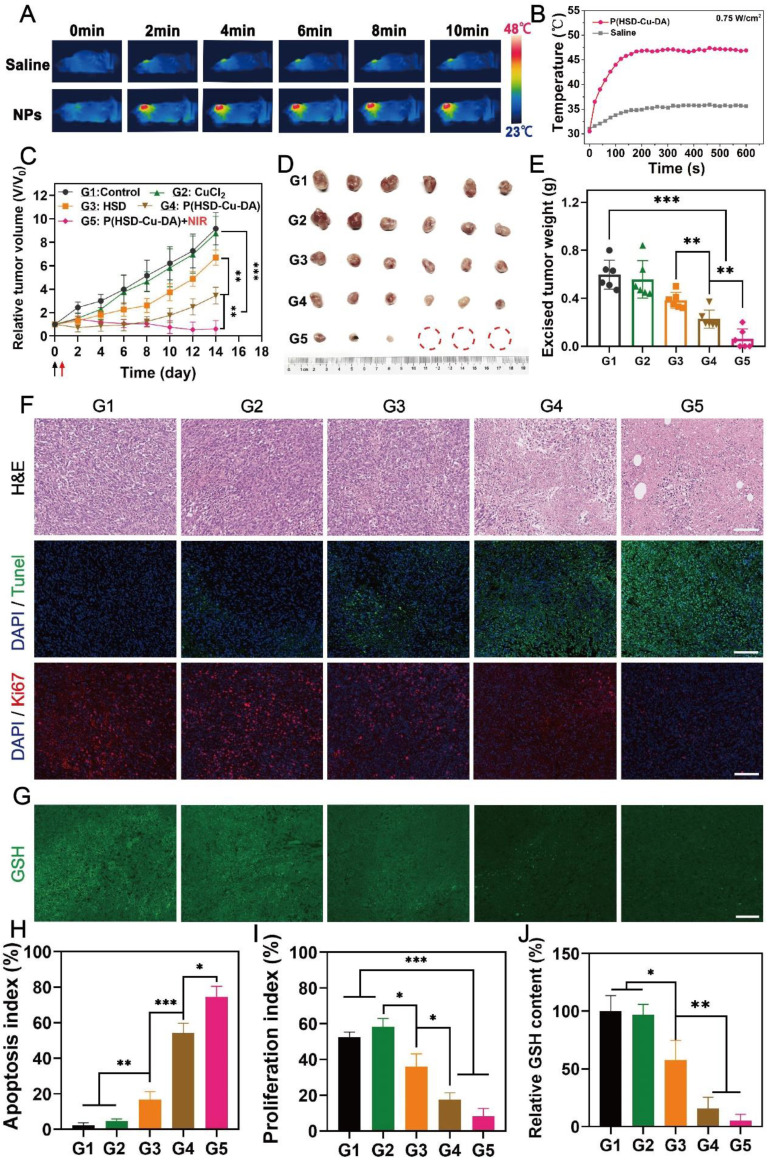
***In vivo* anti-tumor activity of P(HSD-Cu-DA) NPs.** (A) Photothermal imaging (PTI) of 4T1 tumor-bearing mice at 12 h post-injection of saline and P(HSD-Cu-DA) NPs. (B) Real-time temperature changes of the tumor region after NIR irradiation (808 nm, 0.75 W/cm^2^) in different groups. (C) Tumor volume evolvement curves. Black arrow represents intravenous injection and red arrow represent the NIR irradiation. Digital photograph (D) and final tumor weight (E) of the excised tumors by the end of treatment. H&E, TUNEL, Ki67 (F) and GSH staining (G) of the tumor regions after different treatments. Scale bars are 100 μm. (H) Semi-quantification of apoptosis index of the tumor tissue in the TUNEL staining. (I) Semi-quantification of proliferation index of the tumor tissue in the Ki67 staining. (J) Semi-quantification of GSH content in the tumor tissue. G1: Control; G2: CuCl_2_; G3: HSD; G4: P(HSD-Cu-DA); G5: P(HSD-Cu-DA) + NIR. *, P < 0.05; **, P < 0.01; ***, P < 0.001.

Motivated by the superior cell killing ability and high tumor enrichment of P(HSD-Cu-DA) NPs, we further evaluated its anti-tumor potency *in vivo*. NIR light exposure (1 W/cm^2^) was performed after 6 h, 12 h, 24 h of intravenous administration and it was found that the plateau temperature obtained at 12 h was the highest (**Figure S14**), indicating the maximum enrichment of NPs at 12 h, which was consistent with the biodistribution result. Therefore, we finally selected 12 h post-administration as the optimized time for NIR irradiation. Hyperthermia greater than 50°C can effectively ablate tumors, but this may also induce inflammatory responses and thermal damage to nearby normal organs 62. Thus, we managed to maintain the final plateau temperature around 47 ℃ by adjusting the laser power, while the tumor temperature of the mice injected with saline under the same laser exposure had little change (**Figure 6A-B**). Based on these results, the antineoplastic effect of P(HSD-Cu-DA) NPs was studied on 4T1 tumor-bearing mice. The mice were randomly divided into five groups (n=6), namely G1: control, G2: CuCl_2_, G3: HSD, G4: P(HSD-Cu-DA) NPs, and G5: P(HSD-Cu-DA) + Laser. One dosage of drug was intravenously administered for all groups while laser irradiation was applied only once for the last group. As depicted by the variation curves of relative tumor volume in **Figure 6C**, the control group exhibited the fastest tumor growth, which was comparable to the CuCl_2_ group. In the HSD-treated group, there was a slight inhibitory effect on tumor growth relative to the control group, which was attributed to chemotherapy (tumor inhibition rate, TIR=26.9%). Attractively, the group treated with P(HSD-Cu-DA) NPs showed a remarkable tumor inhibition due to the synergistic effect of chemotherapy and CDT (TIR=62.3%). Upon irradiation with NIR laser, the tumor growth was further restrained, and the tumors in three mice were eventually eliminated without recurrence (TIR=93.37%) (**Figure S15**). At the end of the therapeutic process, all mice were euthanized and tumor tissues were harvested. As expected, *ex vivo* tumor results were consistent with tumor suppression curves (**Figure 6D-E**). Subsequently, hematoxylin-eosin (H&E) and Terminal deoxynucleotidyl transferase-mediated dUTP-biotin nick end labeling (TUNEL) stained tumor sections were collected to further evaluate the treatment effect of each group (**Figure 6F**). Of all groups, the mice treated with P(HSD-Cu-DA) NPs under laser irradiation exhibited the highest necrosis and apoptosis ratio (**Figure 6H**), confirming its excellent therapeutic outcome. This result was in excellent agreement with the tumor suppression assay. Furthermore, the proliferation of tumor cells was studied by Ki-67 staining of tumor slices. As shown in **Figure 6F** and **Figure 6I**, the P(HSD-Cu-DA) NPs plus laser group presented the minimum red fluorescence (pseudo-color), indicating an excellent inhibitory effect on tumor cell proliferation. Meanwhile, P(HSD-Cu-DA) NPs alone also significantly depressed tumor cell proliferation relative to control, CuCl_2_ and HSD groups. In *in vitro* experiments, we found that P(HSD-Cu-DA) NPs possessed a good ability to reduce GSH, which was beneficial for CDT. To explore whether the P(HSD-Cu-DA) NPs can regulate redox homeostasis *in vivo*, GSH content in the tumor regions after different treatments were further assessed by using the ThiolTracker Violet fluorescent probe. By observing the GSH probe-stained tumor slices (**Figure 6G**), it was discovered that green fluorescence intensity of P(HSD-Cu-DA) NPs and NIR irradiation group was significantly attenuated than that in control group (**Figure 6J**), indicating the intensive decrease in GSH levels after treatment. While the HSD-treated group exhibited a modest GSH depletion, CuCl_2_ barely depleted GSH in tumor tissues. In addition, we noticed that collagen fibers (stained blue) in tumor tissues were partially degraded after NIR irradiation (**Figure S16**), which was beneficial for the deep penetration of P(HSD-Cu-DA) NPs within the tumor 63, 64. This may also be one of the reasons for the superior therapeutic performance associated with P(HSD-Cu-DA) NPs. In aggregate, all the above results fully demonstrated the excellent synergistic therapeutic outcome of P(HSD-Cu-DA) NPs* in vivo*.

### Safety and biodegradability evaluation of P(HSD-Cu-DA) NPs

**Figure 7:**
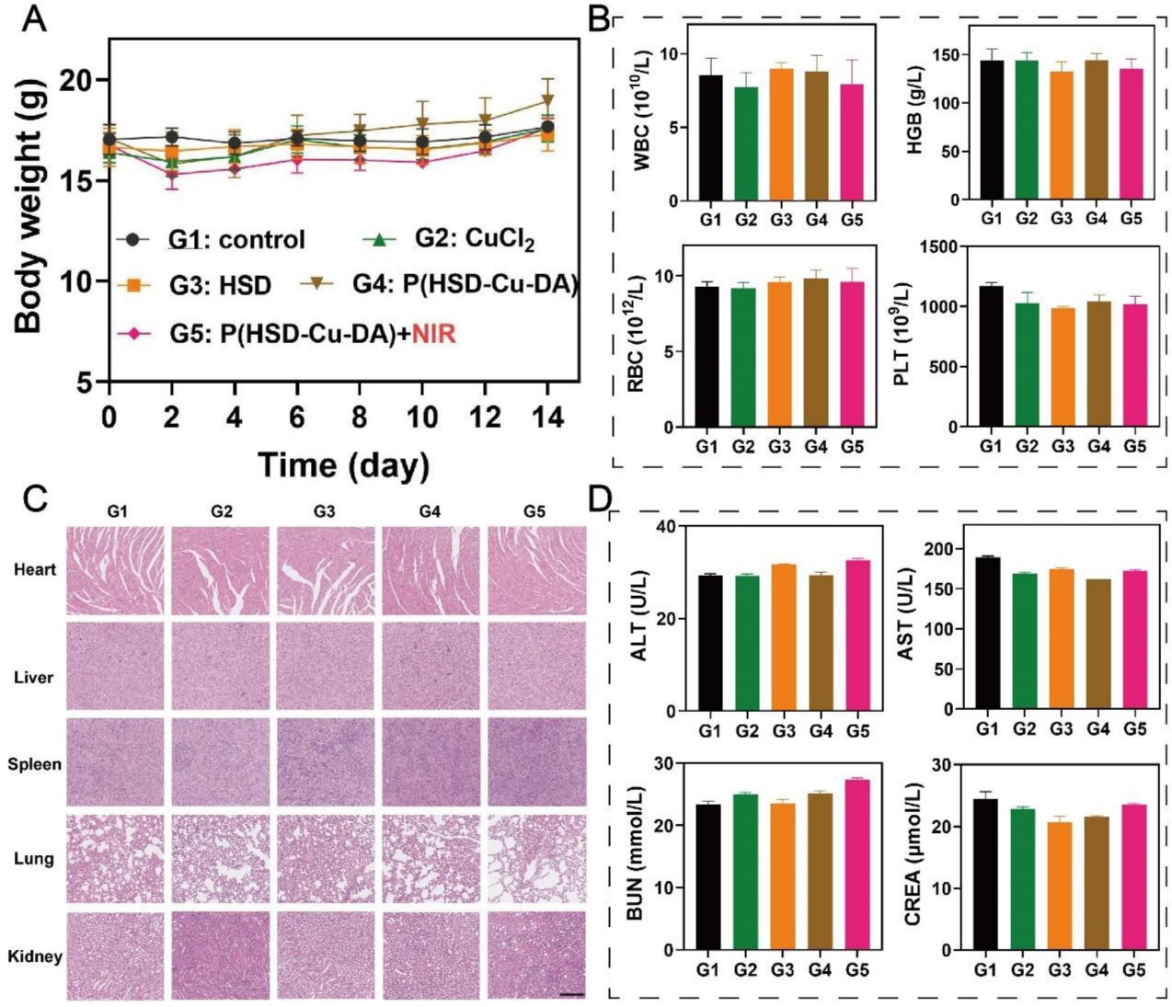
***In vivo* safety assessment of P(HSD-Cu-DA) NPs.** (A) Body weight of mice after various treatments. Routine blood (B) and blood biochemistry (D) analyses of mice in different treatment groups. (C) H&E staining of main organs after different treatments. Scale bar is 100 μm.

The *in vivo* biocompatibility of P(HSD-Cu-DA) NPs was systematically evaluated. During the therapeutic period, the body weight of the mice in all groups showed a slight increasing trend (**Figure 7A**), demonstrating no significant systemic toxicity of our nanotherapeutics. In addition, histological examinations of various main organs (heart, liver, spleen, lung and kidney) of mice after various treatments were carried out to study the potential toxicity. No noticeable tissue damage further demonstrated the biosafety of P(HSD-Cu-DA) NPs (**Figure 7C**). Similarly, the measured indicators for blood routine (WBC, HGB, RBC and PLT) (**Figure 7B**) and blood biochemistry (ALT, AST, BUN and CREA) (**Figure 7D**) were within normal ranges, indicating that P(HSD-Cu-DA) NPs had no apparent side effects during the two weeks of treatment. Besides, P(HSD-Cu-DA) NPs was found to degrade in the presence of H_2_O_2_ (**Figure S17**), mainly attributed to the decomposition of the PDA framework 31. The relatively low H_2_O_2_ content in normal tissues enables the slowly degradation of P(HSD-Cu-DA) NPs. Coincidentally, HES can be degraded *in vivo* by serum α-amylase 38. These characters guarantee the biodegradability of P(HSD-Cu-DA) NPs. Overall, these preliminary data suggest that P(HSD-Cu-DA) NPs hold promise for versatile therapeutic applications with excellent biodegradability and biosafety.

## Conclusions

In summary, we have constructed a biocompatible and stable nanomedicine, P(HSD-Cu-DA) NPs, *via* one-pot coordination and polymerization strategy for self-amplified tumor-specific therapy. Redox-responsive hydroxyethyl starch prodrugs ensure superior circulating stability of nanotherapeutics and enable tumor-specific chemotherapy. Notably, the nanomedicine exhibited an enhanced NIR absorption (8 times of pure PDA at 808 nm) due to the pre-doped Cu^2+^, endowing excellent PAI and PTT as well as thermal-boosted cooperative chemo-/chemodynamic therapy. In the process of activating these therapeutic functions, we demonstrated that both Cu^2+^ reduction and disulfide bond breaking engendered GSH consumption, which together amplify the oxidative stress of cancer cells. Both *in vitro* and *in vivo* results demonstrate the excellent cooperative therapeutic outcome of P(HSD-Cu-DA) NPs against tumors, with minimal toxicity to normal cells and tissues. Both PDA framework and HES are biopolymer in this nanoplatform, guaranteeing long-term safety. The goal of precision medicine is to maximize the effectiveness of treatment while minimize side effects. For our nanoplatform, we integrate tumor-specific CDT, PTT and redox-sensitive chemotherapy to minimize side effects, and more importantly, these therapeutic components do not exist in isolation but rather complement each other to ultimately achieve optimum tumor therapy. Given the excellent biosafety, superior stability and TME-activated therapeutic behavior, this nanoplatform represents an insightful example of effective cancer therapy with significant potential for clinical translation.

## Methods

### Materials and reagents

**Chemicals.** Hydroxyethyl starch (HES) with average molecular weight (Mw) 130 kDa, molar substitution of hydroxyethyl 0.4 was gift from Life Science & Technology Co., Ltd. (Wuhan, China). Doxorubicin (99%) was bought from Beijing Huafenglianbo Technology Co., Ltd. (Beijing, China). Copper (II) chloride dihydrate (CuCl_2_^.^2H_2_O, 99.99%), dopamine hydrochloride (98%), copper standard solution (500 ppm), methylene blue, dithiothreitol (DTT) and reduced glutathione (GSH, 98%) were purchased from Sigma-Aldrich. Ammonia solution (NH_3_.H_2_O, 25-28%), methanol, dimethyl sulfoxide (DMSO), tween-80 and hydrogen peroxide (H_2_O_2_) were purchased from SinopharmChemical Reagent Co.Ltd, (Shanghai, China). Ultrapure water (Millipore Milli-Q grade, 18.2 MΩ) was used in all the experiments. All chemicals were analytical grade and used directly without further purification.

**Biological reagents**. GSH and GSSG Assay Kit and Cytotoxicity Assay Kit were bought from Beyotime. Annexin V-APC/7-AAD apoptosis kit was purchased from Multisciences (LianKe) Biotech, Co., Ltd. 2, 7-Dichlorodi-hydrofluorescein diacetate (DCFH-DA), Cell Counting Kit-8 (CCK-8) and Calcein-AM/PI double stain kit was purchased from Yeasen Biotechnology Co., Ltd. RPMI 1640 medium and fetal bovine serum (FBS) were purchased from Gibco BRL/Life Technologies, Grand Island, NY, USA.

### “One pot” synthesis of P(HSD-Cu-DA) NPs

Redox-responsive prodrug HES-SS-DOX (DOX loading capacity, 4.86%) was synthesized according to previous work of our lab 37. For the synthesis of P(HSD-Cu-DA) NPs, 0.02 mmol of dopamine hydrochloride and HES-SS-DOX were foremost dissolved in 8.9 mL deionized water to achieve DA/DOX molar ratio of 1:0.1, 0.2, 0.3, 0.4, 0.5. Then, the aqueous 20 mM solution of CuCl_2_ (1 mL) was dropwise added to the mixed solution. After 1 h of coordination at room temperature, 0.1 mL ammonia solution was quickly injected into the above solution to start polymerization. The reaction was allowed overnight under stirring, resulting a P(HSD-Cu-DA) solution, which was purified by dialysis (MWCO: 300 kDa) for three days, followed by lyophilization. The preparation of P(Cu-DA) follows the same procedure without HES-SS-DOX.

As a control, pure polydopamine (PDA) NPs with same amount of monomer (DA) was synthesized according to previous literature 65.

The drug content in P(HSD-Cu-DA) NPs was calculated as follows:

DOX loading content (mass%) = 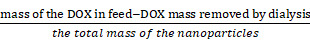 ×100%

### Apparatus

The hydrodynamic diameter and Zeta-potential profiles were acquired on a dynamic light scattering instrument (DLS, Zetasizer Nano ZS90, Malvern, UK). The morphology of NPs was characterized by transmission electron microscopy (TEM, HT7700 HITACHI Co., Japan, accelerating voltage: 120 kV). The micro-area element analysis was conducted on field emission transmission electron microscopy (FTEM, Talos F200X, FEI Co., Netherlands, accelerating voltage: 200 kV). Copper content was determined by inductively coupled plasma optical emission spectrometer (ICP-OES, PerkinElmer Ltd., Co., USA). Copper valence was measured by X-ray photoelectron spectroscopy (AXIS-ULTRA DLD-600 W, Shimadzu-Kratos Co., Japan). Characteristic groups of the samples were verified using a Fourier transform infrared spectroscopy (FTIR, VERTEX 70, Bruker Co., Germany). The absorption spectra were collected using a UV-Vis spectrophotometer (Lambda 35, PerkinElmer Instruments Co., Ltd., Shanghai, China). The cellular behavior was observed using Confocal laser scanning microscope (CLSM, Olympus, FV3000).

### Cell lines and animal

4T1, Panc02, B16-F10 cancer cells, and HUVECs were purchased from BeNa Culture Collection, China. The cells were cultured in RPMI 1640 medium supplemented with 10% fetal bovine serum (FBS) and 1% penicillin/streptomycin under humidified conditions with 5% CO_2_ and 37 ℃.

BALB/c (female, SPF, 5 weeks) were purchased from Beijing Vital River Laboratory Animal Technology Co. Ltd. All animal handing procedures were performed in accordance with the internationally accepted principles and Guidelines for the Care and Use of Laboratory Animals of Huazhong University of Science and Technology. Experimental protocols were approved by the Institutional Animal Ethical Committee of the Huazhong University of Science and Technology. The mice were kept in specific pathogen-free environment, and had access to food and water ad libitum.

### Stability test

The lyophilized P(HSD-Cu-DA) NPs were redispersed in three different media, that is, saline, PBS buffer (0.01 M, pH7.4) and 10% (v/v) FBS. The stability of NPs was evaluated by monitoring size changes in different media for a week. The long-term stability (30 day) of P(HSD-Cu-DA) and P(Cu-DA) in PBS buffer (0.01 M, pH7.4) was also tested.

### Measurement of photothermal performance

To study the effect of concentration on temperature elevation, different concentrations of P(HSD-Cu-DA) NPs solutions (0-1.2 mg/mL) were prepared, and irradiated by an 808-nm NIR laser (1 W/cm^2^) for 10 min. Compared to pure PDA, the coordination of Cu significantly improves the absorption of P(HSD-Cu-DA) in the near-infrared region. Herein, the temperature rise of the PDA (with same amount of DA) was measured for comparison. The photothermal stability of P(HSD-Cu-DA) was also tested by recording three cycles of heating and cooling period. The temperature of the solutions was monitored by a thermal imager, and recorded every 20 seconds. The photothermal conversion efficiencies (η) of P(HSD-Cu-DA) and PDA were calculated according to previous literatures 31, 66.

### Detection of ·OH production

Methylene blue (MB) was employed as a probe to detect ·OH generated by P(HSD-Cu-DA) NPs. In brief, P(HSD-Cu-DA) NPs (1 mM) and GSH (1 mM) were firstly mixed in buffer solution (pH 6.5), followed by the supplementation with MB (10 μg/mL) and H_2_O_2_ (10 mM). The other three control groups were (1) MB, (2) MB+H_2_O_2_, (3) P(HSD-Cu-DA) NPs+MB+H_2_O_2_. Then, the above mixed solutions were stirred at room temperature (25 ℃) for 2 h. After centrifugation, the absorbance of MB in supernatant were recorded by a UV-vis spectrophotometer.

For investigation of GSH content on ·OH production, the conditions were similar to the above, except the supplementation with different amounts of GSH (0-4 mM). To study the influence of hyperthermia on ·OH generation, mixed solutions of 1mM P(HSD-Cu-DA), 10 μg/mL MB, 10 mM H_2_O_2_, and 1 mM GSH were allowed to incubate with/without NIR irradiation for 30 min. The absorbance change of MB was measured.

### Extracellular GSH depletion

The variation of GSH content was measured by Ellman's reagents (DTNB). Firstly, 10 mg DTNB was dissolved in 1 mL methanol to obtain a stock solution of DTNB (10 mg/mL), storing in a dark place at low temperature for later use. Then, P(HSD-Cu-DA) ([Cu]=1 mM) was mixed with GSH (1 mM) in PBS solution at room temperature. At different time points, 0.1 mL of the mixed solution was pipetted into 0.9 mL PBS solution, followed by adding 4 μL DTNB stock solution. After incubation for 5 min, the absorbance spectrum of the mixture was measured.

### *In vitro* stimulus-responsive drug release

The release profile of DOX from P(HSD-Cu-DA) NPs was performed using dialysis method 67. Briefly, 1 mL of P(HSD-Cu-DA) solution (DOX=0.15 mg) was packaged into a dialysis tube (MWCO: 3500 Da). The tube was then immersed within 30 mL of release solution and shaken at 37 ℃ for a period of time. The release solutions were set as follows, (1) PBS buffer (0.01 M, pH 7.4, 0.5% Tween-80), (2) PBS buffer (0.01 M, pH 5.0, 0.5% Tween-80), (3) PBS buffer (0.01 M, pH 7.4, 0.5% Tween-80) with 10 mM DTT. At each preset time point, 0.2 mL of the external release solution was collected, while 0.2 mL fresh release solution was supplemented to keep the volume constantly.

Likewise, to determine hyperthermia-triggered drug release behavior, 1 mL of P(HSD-Cu-DA) solution (DOX=0.15 mg) was transferred into a dialysis tube and then immersed in 30 mL of PBS buffer containing 10 mM DTT/GSH. At three different time point, the sample was carefully taken out and irradiated with an 808 nm NIR laser (1 W/cm^2^) for 10 min, and then put back into dialysis tube to continue the following release experiments. The tube without NIR irradiation was as control.

Each release experiments had three replicates. The concentration of released DOX was determined by a multi-mode microplate reader with excitation wavelength at 483 nm and emission wavelength at 556 nm.

### Cu^2+^ release from P(HSD-Cu-DA) NPs

1 mL of P(HSD-Cu-DA) solutions ([Cu]=64 ppm) were prepared and placed in dialysis tubes. These tubes were then immersed in different release buffers (30 mL) which was PBS buffer (0.01 M, pH 7.4), PBS buffer (0.01 M, pH 5.0) and PBS buffer (0.01 M, pH 7.4) with 10 mM DTT. At predetermined time intervals, 5 mL of release buffer was taken out and 5 mL of corresponding fresh buffer was replenished. The Cu concentration in release buffers were measured by ICP-OES. The experiments were proceeded in triplicate.

### Cellular uptake of P(HSD-Cu-DA) NPs

ICP-OES was applied to study the uptake behavior of P(HSD-Cu-DA) NPs by 4T1 cells. In brief, cells were cultured in 12-well plate at a density of 1×10^5^ cells per well. After incubation with P(HSD-Cu-DA) NPs ([Cu]=1.66 μg/mL) for different time, the cells were digested with trypsin and rinsed 3 times with PBS. After cell counting, the precipitate was retained by centrifugation and subsequently digested with HNO_3_ and HClO_4_ (*v*/*v*=4:1) for ICP-OES testing.

### Detection of intracellular ROS generation

Fluorescence probe DCFH-DA was employed to detect ROS. Briefly, 4T1 cells were randomly seeded in four confocal dishes at a density of 1×10^5^ cells/well for 12h before further manipulation. Then, the cells were cultured with DCFH-DA (10 μM, dilute with RPMI 1640 medium) for 30 minutes. After rinsing with PBS for three times, the cells were treated with P(HSD-Cu-DA) NPs ([Cu] = 6 μg/mL) for 6h. After that, the cells were exposed to an 808-nm laser for 5 min (1 W/cm^2^) and gently washed twice with PBS to remove uningested NPs. The other three groups were (1) blank group incubated only with RPMI-1640 medium, (2) control group cultured with CuCl_2_ solution ([Cu]= 6 μg/mL), and (3) experimental group incubated with P(HSD-Cu-DA) ([Cu]=6 μg/mL). Finally, the fluorescence images ware observed by CLSM (λ_ex_= 488 nm, λ_em_= 525 nm). All the experiments were done in triplicate.

### Intracellular GSH assay

For GSH detection, 4T1 cells were seeded into 12-well plate (1×10^5^ cells/well) and incubated at 5% CO_2_, 37 ℃ until adherence. Afterwards, the cells were treated with PBS, CuCl_2_, HSD and P(HSD-Cu-DA) ([Cu]=16 μg/mL). After incubating for 6 h, the GSH content was measured by a GSH and GSSG Assay Kit (Beyotime) according to the manufacturer's instructions. In addition, changes in intracellular GSH content after treatment with different concentrations of P(HSD-Cu-DA) were also tested according to similar procedures.

### *In vitro* cytotoxicity assessment

For cytotoxicity evaluation, 4T1 cells and HUVECs were seeded on a 96-well plate (8000 cells per well), respectively, and incubated in 5% CO_2_ at 37 ℃ overnight. The cells were then treated with different concentrations of HSD or P(HSD-Cu-DA) for 48 h. Later, the medium was discarded and washed with PBS twice. After incubated with prepared MTT solution for another 4 h, the supernatant was replaced with DMSO to dissolve the formed crystals. Finally, the absorbance at 492 nm of each well was measured using a microplate reader. The cell viability was normalized by control group cultured with free medium alone. All results were averaged from five replications.

To verify hyperthermia-mediated cytotoxicity enhancement, 4T1 cells were cultured with different concentrations of P(HSD-Cu-DA) for 6h, and then irradiated with or without an 808 nm laser (1 W/cm^2^) for 5 min. After another 42h incubation, the cell viability was acquired according to the above-mentioned MTT assay.

### Live/dead staining

4T1 cells were seeded in confocal dishes (1×10^5^ cells per well) and incubated overnight. After which, the medium was discarded and replenished with fresh medium containing HSD and P(HSD-Cu-DA) (the DOX concentration is 8 μg/mL). After co-culture for 6 h, the cells were treated with or without NIR laser (808 nm, 1 W/cm^2^) for 5min. Thereafter, the cells were cultured for another 42 h, followed by staining with Calcein-AM/PI for observing cell viability by CLSM.

### Cell apoptosis and necrosis assay

Annexin V-APC/7-AAD apoptosis kit was used to detect the cell apoptosis and necrosis. 4T1 cells were seeded in 12-well plates and incubated until adherence. Then the cells were exposed to different treatments including (1) PBS, (2) HSD, (3) P(HSD-Cu-DA) and (4) P(HSD)-Cu-DA + 5 min NIR irradiation (an equivalent DOX amount of 8 μg/mL). After co-culture for 24 h, the cells were harvested and stained with Annexin V-APC and 7-AAD, subsequently analyzing by flow cytometry.

### Tumor model

4T1 tumor model were established by subcutaneous injection of 4T1 cells (1×10^6^ cells in 100 μL sterile PBS) into the right flank of the BALB/c mice. The tumorous growth was monitored daily. The length (L) and width (W) of tumors were measured with a vernier caliper, and then the tumorous volume (V) was calculated by the following formula: V= L×W^2^/2^8^. *In vivo* experiments were conducted when the tumorous volume reached about 80 mm^3^.

### *In vitro* and *in vivo* Photoacoustic (PA) imaging

For *in vitro* PA experiments, different concentrations of P(HSD-Cu-DA) NPs (0, 0.8, 1.6, 2.4, 3.2 and 4 mg/mL) dissolved in PBS were used for PA signal detection. The linearity between PA intensity and NPs concentration was also assessed.

To evaluate PA imaging ability of P(HSD-Cu-DA) NPs *in vivo*, 4T1 tumor-bearing mice were treated with P(HSD-Cu-DA) NPs with intravenous injection. After 12 h, the mice were anesthetized with pentobarbital sodium and fixed on a tailored holder. Subsequently, the tumor sites were scanned by a PA imaging system to collect PA signals. The PA signal collected before administration was used as a control. The excitation wavelength used in this experiment was set to 744 nm, and the laser energy per pulse was 140 nJ.

### Pharmacokinetic and biodistribution study

4T1 tumor-bearing mice (n=3) were employed to study pharmacokinetic of P(HSD-Cu-DA) NPs. Briefly, free CuCl_2_ solution or P(HSD-Cu-DA) (Cu equivalent of 1.75 mg/kg) was systematically injected via tail vein. At desired time points (0.25 h, 0.5 h, 1 h, 2 h, 5 h, 12 h, 24 h and 48 h), the blood samples (10 μL) were extracted and digested overnight. Subsequently, the concentration of Cu in each sample was measured by ICP-OES.

For biodistribution study, P(HSD-Cu-DA) NPs with Cu concentration of 1.75 mg/kg were injected into 4T1 tumor-bearing mice (n=15) though tail vein. These mice were sacrificed, and their tumors and other main organs including heart, liver, spleen, lung and kidney were collected at 6 h, 12 h, 24 h, 3 day, and 7 day post injection. After weighing, the excised tumors/tissues were digested with HNO_3_ and HClO_4_ (*v*/*v*=4:1) at 300℃ until to form a clear solution. The Cu content was determined by ICP-OES.

### *In vivo* photothermal imaging (PTI)

To evaluate PTI performance *in vivo*, the tumor-bearing mice were injected with P(HSD-Cu-DA) NPs (Cu concentration of 1.75 mg/kg, 200 μL). At 12 h post injection, the mice were anesthetized and the tumor areas were exposed to a NIR laser (0.75 W/cm^2^, spot diameter 1cm) for 10 min. The real-time temperature was monitored every 20 seconds using a NIR thermal camera.

### *In vivo* tumor therapy assessment and histological staining

4T1 tumor-bearing mice were randomly divided into five groups (six mice per group), and then intravenously injected with (1) saline, (2) CuCl_2_, (3) HSD, (4) P(HSD-Cu-DA) and (5) P(HSD-Cu-DA) + NIR laser (injection dose= 200 μL, the equivalent concentrations of DOX and Cu were 3.52 mg/kg and 1.75 mg/kg). For Group 5, 12 h post administration, the NIR laser continued illuminate the tumor site for 10 min with power density of 0.75 W/cm^2^ and spot diameter of 1 cm. One dosage of drug was administered for all groups while laser irradiation was also applied for one time for the last group. The tumor dimensions and body weight were recorded every two days. The tumor inhibition rate (TIR) for each group was calculated using the following equation: inhibition ratio=V_c_ - V_e_ / V_c_×100%, where V_c_ represents the tumor volume of the blank group and V_e_ represents the tumor volume of the experimental group. At the end of the treatments, the experimental mice were euthanized and the tumors were gathered, photographed and weighted. The therapeutic effect was also evaluated by histological assay. Excised tumors were fixed, embedded, and then stained with Hematoxylin and Eosin (H&E), Ki67, and terminal deoxynucleotidyl transferase dUTP nick end labeling (TUNEL). GSH and Masson staining was conducted on tumors collected 24h after treatments.

### Safety evaluation

For histological evaluation, the major organs (heart, liver, spleen, lung and kidney) of different groups were also harvested and subjected to H&E staining. Besides, blood samples of treated mice were gathered for blood routine test (WBC, HGB, RBC and PLT) and blood biochemistry analysis (ALT, AST, BUN and CREA).

### Statistical analysis

All the data in the present study were processed and presented as mean ± standard deviation (SD). The semi-quantification of fluorescence was conducted with image J 1.53c. The statistical significances between different groups were assessed with one-way ANOVA and a student's t-test. All statistical calculations were conducted on GraphPad Prism 8.0. (n.s., not significant; *P < 0.05, significant; **P < 0.01, moderately significant; ***P < 0.001, highly significant).

## Supplementary Material

Supplementary figures.
