# Supplementary material for: Tumor-specific activatable biopolymer nanoparticles stabilized by hydroxyethyl starch prodrug for self-amplified cooperative cancer therapy
Source: Theranostics. 2022 Jan 1;12(2):944–62. doi: 10.7150/thno.67572 (PMC8692913; doi:10.7150/thno.67572)
Supplement: Supplementary file 1 — Supplementary figures. [file thnov12p0944s1.pdf]

Supporting Information for

Tumor-specific activatable biopolymer nanoparticles stabilized by hydroxyethyl starch prodrug for self-amplified cooperative cancer therapy

Yuxuan Xiong[a,#], Zibing Wang[a,#], Qiang Wang[a], Qingyuan Deng[a], Jitang Chen[a], Jianshuang Wei[b], Xiaoquan Yang[b], Xiangliang Yang[a,c,d,f], Zifu Li[a,c,d,e,g,*]

[a] National Engineering Research Center for Nanomedicine, College of Life Science and Technology, Huazhong University of Science and Technology, Wuhan, 430074, P. R. China

[b] Key Laboratory of Biomedical Photonics (HUST), Ministry of Education, Huazhong University of Science and Technology, Wuhan 430074, P. R. China

[c] Key Laboratory of Molecular Biophysics of Ministry of Education, College of Life Science and Technology, Huazhong University of Science and Technology, Wuhan, 430074, P. R. China

[d] Hubei Key Laboratory of Bioinorganic Chemistry and Materia Medical, Huazhong University of Science and Technology, Wuhan, 430074, P. R. China

[e] Hubei Engineering Research Center for Biomaterials and Medical Protective Materials, Huazhong University of Science and Technology, Wuhan, 430074, P. R. China

[f] GBA Research Innovation Institute for Nanotechnology, Guangdong, 510530, P. R. China

[g] Wuhan Institute of Biotechnology, High Tech Road 666, East Lake high tech Zone, Wuhan, 430040, P. R. China

[#] These authors contribute equally.

* Correspondence and requests for materials should be addressed to ZFL (email: zifuli@hust.edu.cn).

Supplementary Figures

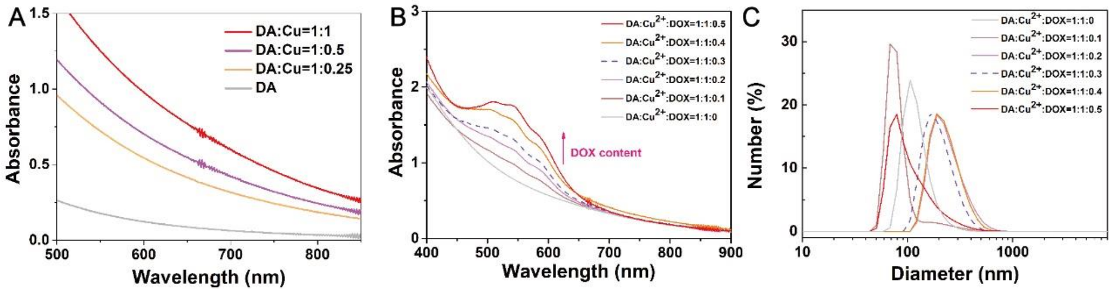

Figure S1. (A) Absorption spectra of P(Cu-DA) obtained in different ratios. (B) Absorption spectra of P(HSD-Cu-DA) obtained in different ratios. (C) DLS results for P(HSD-Cu-DA) NPs acquired with different ratios.

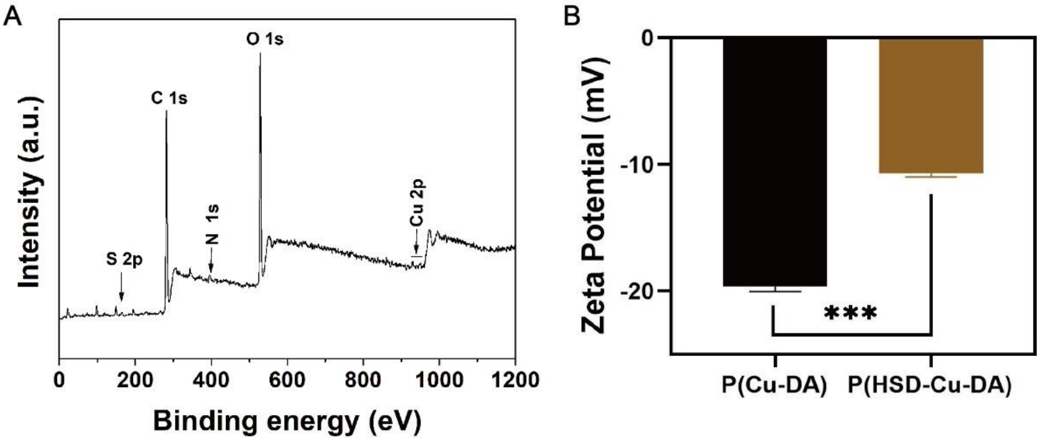

Figure S2. (A) XPS survey spectrum of P(HSD-Cu-DA) NPs. (B) Zeta potential of P(Cu-DA) and P(HSD-Cu-DA) NPs.

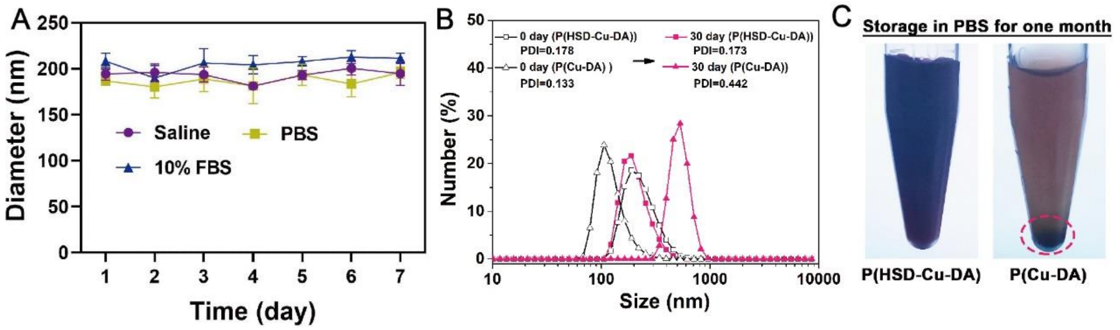

Figure S3. (A) Stability test of P(HSD-Cu-DA) NPs in different media (n=3). (B) Long-term stability of P(Cu-DA) and P(HSD-Cu-DA) NPs. (C) Digital photo of P(HSD-Cu-DA) and P(Cu-DA) NP solutions after storage in PBS for one month.

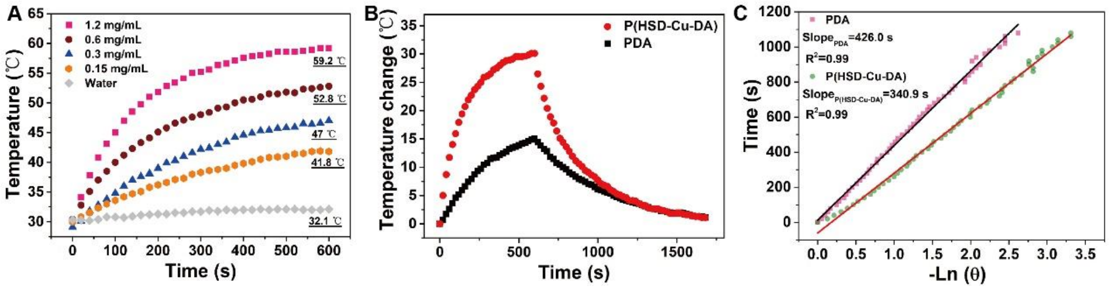

Figure S4. (A) Temperature rising curves for different concentrations of P(HSD-Cu-DA) NPs solution. (B) Temperature rise and fall curves for P(HSD-Cu-DA) and PDA NPs. (C) Linear relationship between -Ln (θ) and time (s) obtained from the cooling curves.

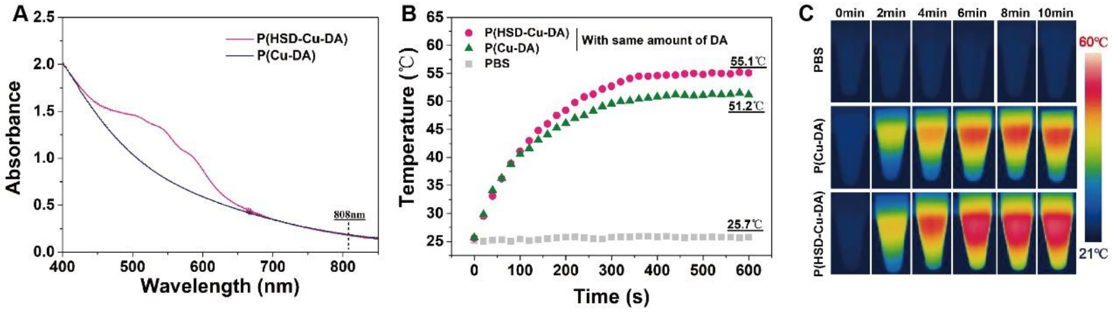

Figure S5. (A) UV-Vis spectra of P(Cu-DA) and P(HSD-Cu-DA) with same amount of DA and Cu^{2+}. Temperature changes (B) and corresponding photothermal images (C) of P(Cu-DA) and P(HSD-Cu-DA) solution under NIR irradiation (808nm, 1W/cm^2).

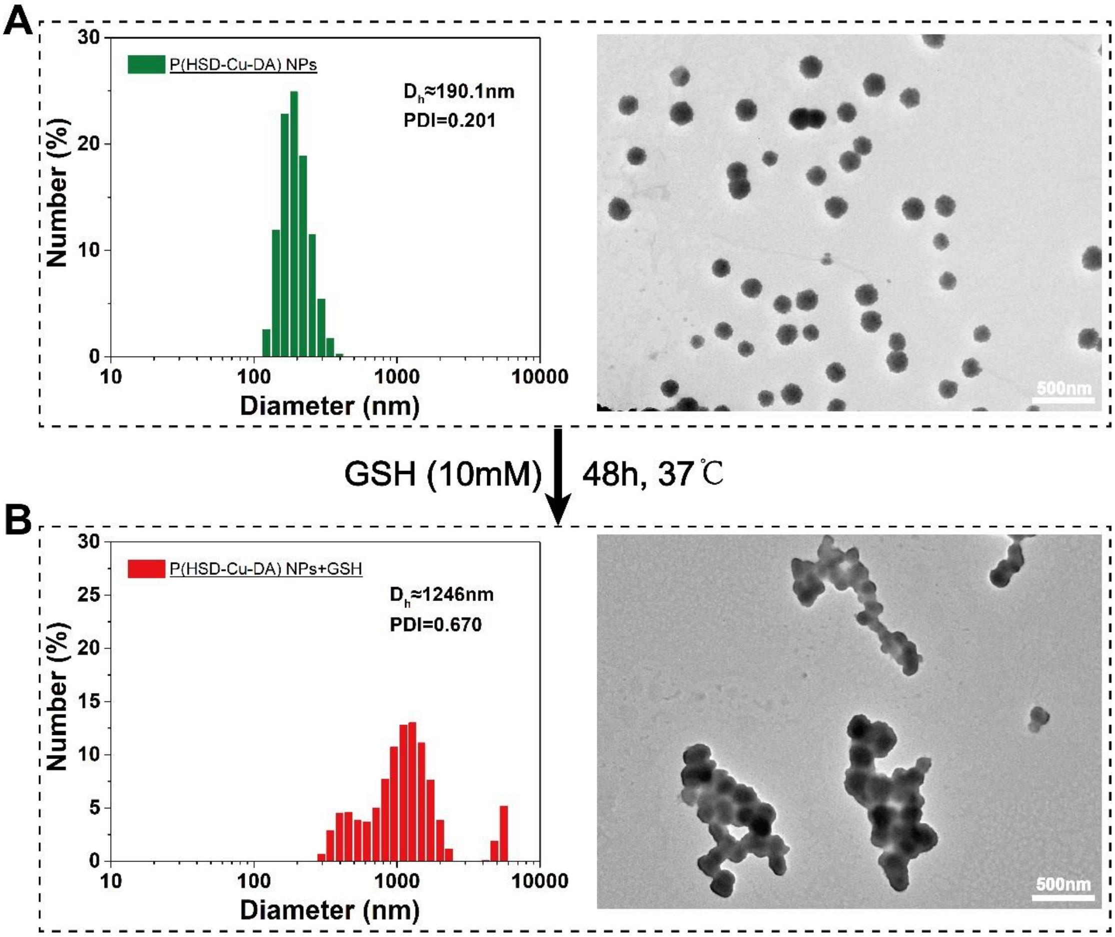

Figure S6. (A) Size distribution and TEM image of P(HSD-Cu-DA) NPs. (B) Size distribution and TEM image of P(HSD-Cu-DA) NPs treated with GSH (10mM) for 48h.

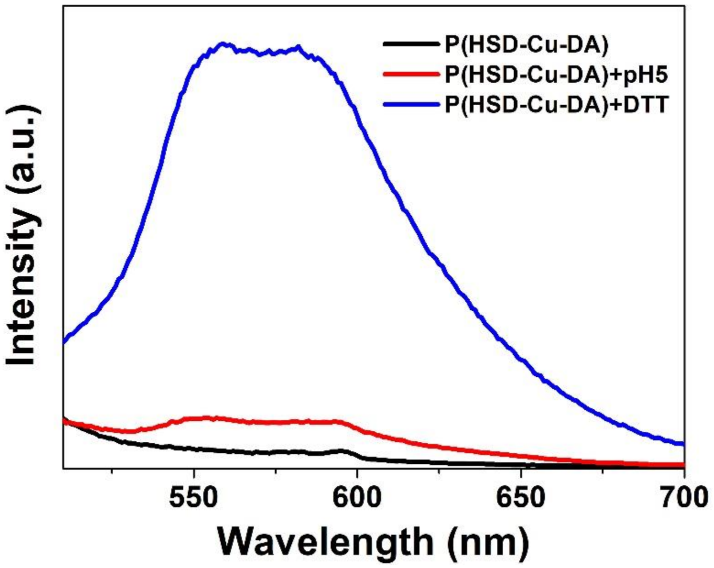

Figure S7 FL emission spectra of P(HSD-Cu-DA) NPs under different conditions (excitation wavelength = 490nm).

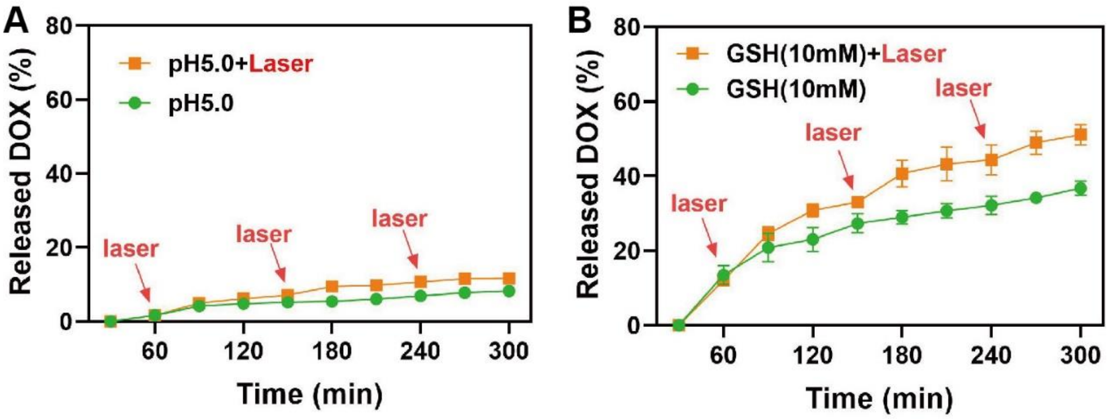

Figure S8. In vitro release profiles of P(HSD-Cu-DA) NPs with/without NIR irradiation under pH5 condition (A) or in the presence of GSH (B).

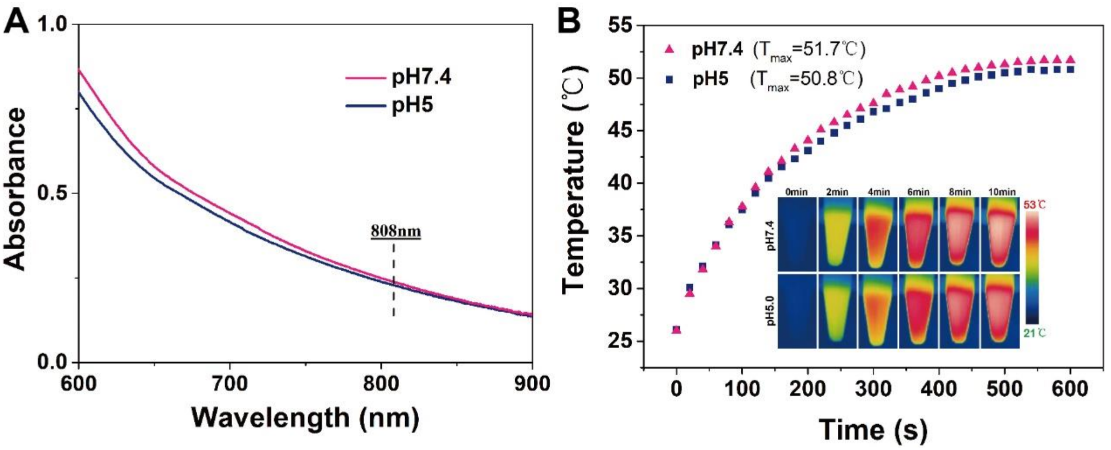

Figure S9. (A) UV-Vis spectra of P(HSD-Cu-DA) NPs after incubation at different pH conditions for 48h. (B) Temperature changes and corresponding photothermal images of P(HSD-Cu-DA) solution upon NIR irradiation (808nm, 1W/cm^2) after incubation at different pH conditions.

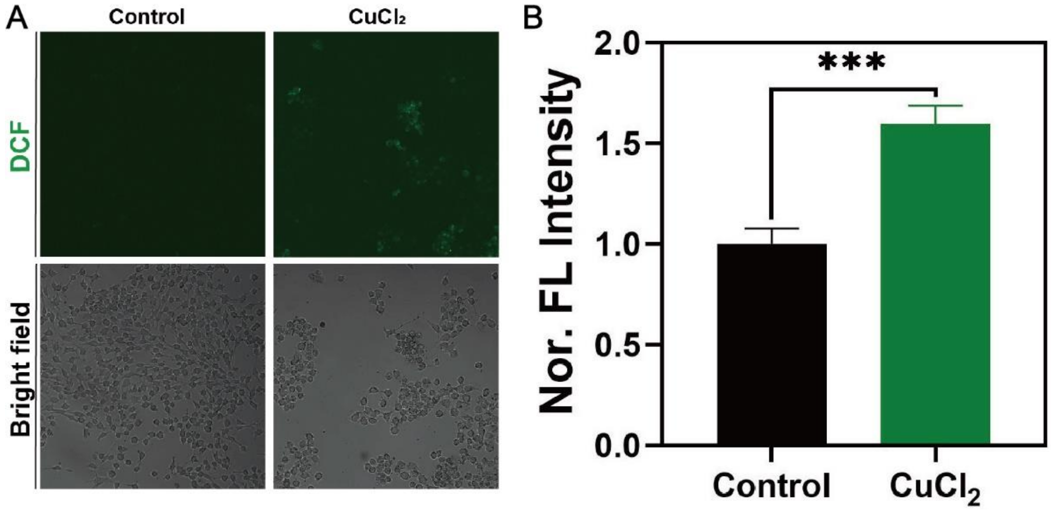

Figure S10. CLSM images (A) and semi-quantification (B) of 4T1 cells with ROS probe in different treatments.

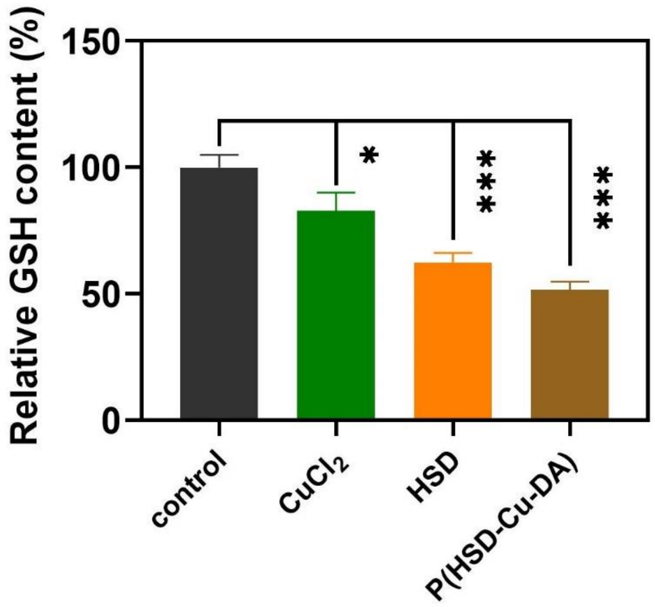

Figure S11. Relative intracellular GSH content in 4T1 cells after different treatments.

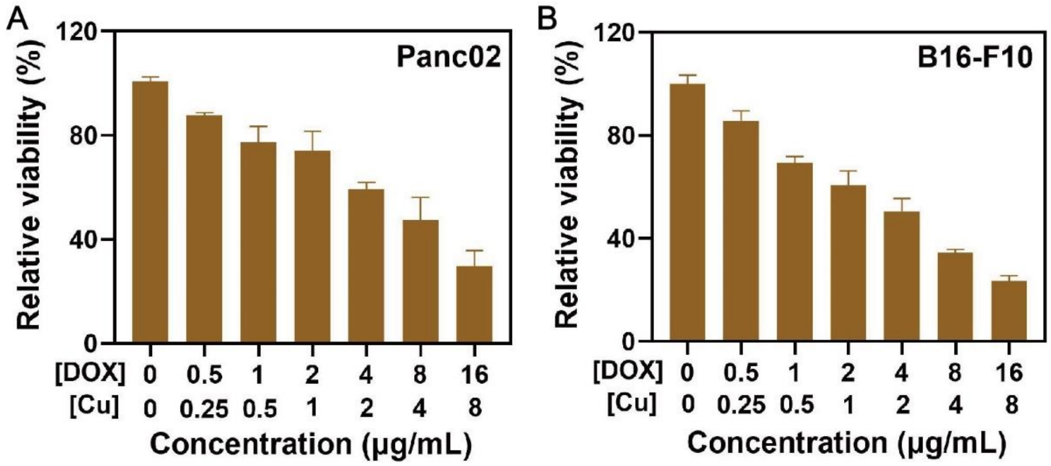

Figure S12. Cytotoxicity of P(HSD-Cu-DA) NPs on Panc02 cells (A) and B16-F10 cells (B).

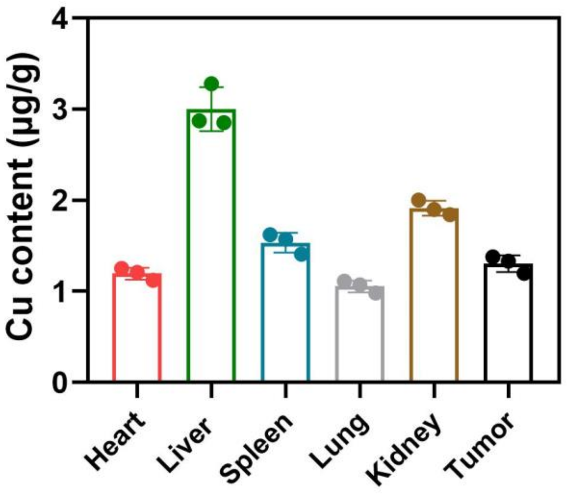

Figure S13. Cu contents in different organs and tumor of blank mice (n=3).

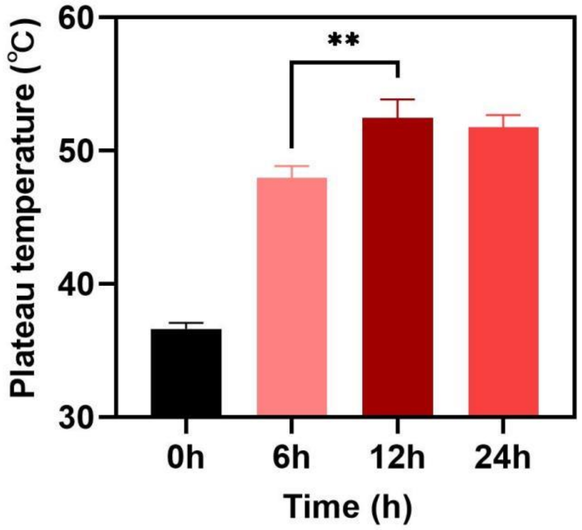

Figure S14. The plateau temperatures obtained by NIR irradiation (1W/cm^2) after different times of intravenous administration.

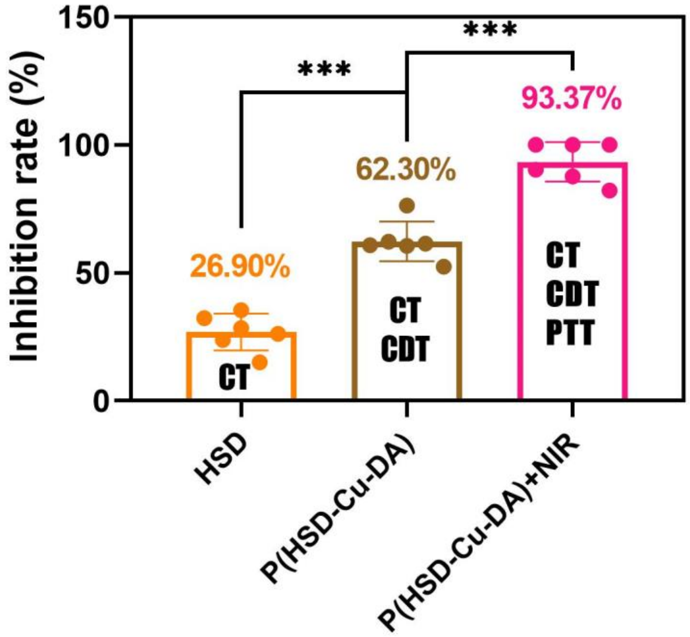

Figure S15. The tumor inhibition rate (TIR) of different groups after 2-week therapy.

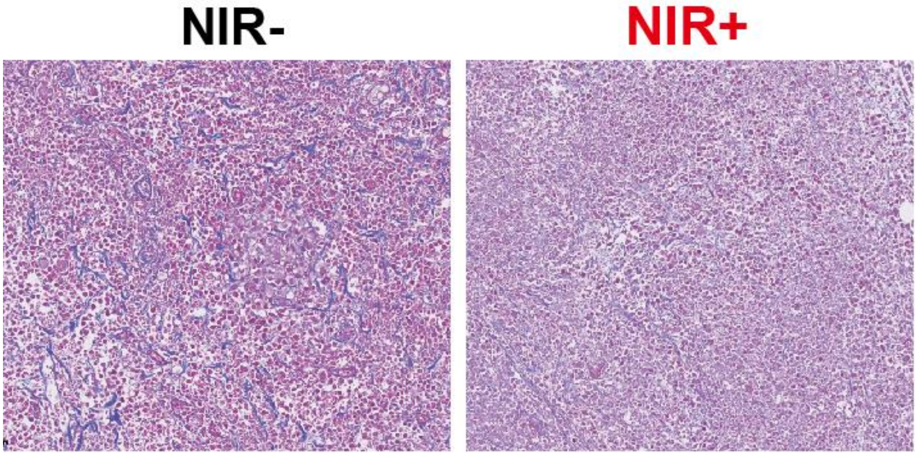

Figure S16. Masson staining images with or without NIR exposure.

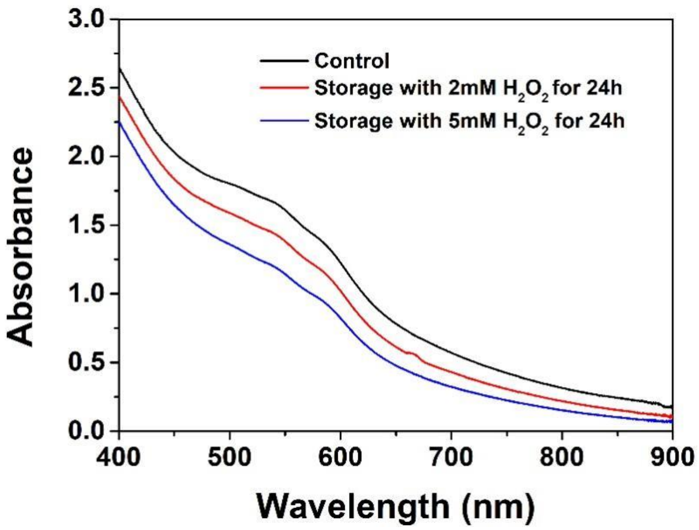

Figure S17. UV-Vis spectra of P(HSD-Cu-DA) NPs after incubation with different concentrations of H_2O_2 for 24 h.
